# Different roles of Rac1 in the acquisition and extinction of methamphetamine-associated contextual memory in the nucleus accumbens

**DOI:** 10.7150/thno.34655

**Published:** 2019-09-21

**Authors:** Jinlan Zhao, Li Ying, Yutong Liu, N Liu, Genghong Tu, Mengjuan Zhu, Yue Wu, Bin Xiao, Liuzhen Ye, Juan Li, Fukun Guo, Lin Zhang, Huijun Wang, Lu Zhang

**Affiliations:** 1 Key Laboratory of Functional Proteomics of Guangdong Province, Key Laboratory of Mental Health of the Ministry of Education, School of Basic Medical Sciences, Southern Medical University, Guangzhou 510515, China; 2 Department of Histology and Embryology, Guangdong Provincial Key Laboratory of Tissue Construction and Detection, School of Basic Medical Sciences, Southern Medical University, Guangzhou 510515, China; 3 School of Forensic Medicine, Southern Medical University, Guangzhou 510515, China; 4 Institute of Comparative Medicine & Laboratory Animal Center, Southern Medical University, Guangzhou 510515, China; 5 Division of Experimental Hematology and Cancer Biology, Children's Hospital Research Foundation, Cincinnati, OH, USA

**Keywords:** Rac1, nucleus accumbens, spine plasticity, METH addiction, drug reward memory

## Abstract

**Rationale:** Repeated methamphetamine (METH) exposure induces long-term cognitive deficits and pathological drug-associated memory that can be disrupted by manipulating memory reconsolidation and extinction. The nucleus accumbens (NAc) is the key region of the brain reward system and predominantly consists of two subtypes of medium spiny neurons (MSNs) based on the expression of D1 or D2 dopamine receptors (D1-MSNs or D2-MSNs). Spine structural plasticity in the NAc is critical for the acquisition, reconsolidation and extinction of drug-associated memory. However, the molecular mechanisms underlying METH-associated memory and spine remodelling in each type of MSNs in the NAc remain unknown. Here, we explored whether Rac1 in the NAc mediates METH-associated contextual memory and spine remodelling.

**Methods:** Pharmacological and genetic manipulations of Rac1 were used to investigate its role during the acquisition, reconsolidation and extinction of METH-associated contextual memory. Recombinant adeno-associated viruses expressing mCherry under the control of the dopamine D1 receptor gene promoter (Drd1-mCherry) or dopamine D2 receptor gene promoter (Drd2-mCherry) were used to specifically label D1-MSNs or D2-MSNs.

**Results:** Using viral-mediated gene transfer, we demonstrated that decreased Rac1 activity was required for the acquisition of METH-associated contextual memory and the METH-induced increase in thin spine density, whereas increased Rac1 signalling was important for the extinction of METH-associated contextual memory and the related elimination of thin spines. Moreover, the increase of dendritic spines was both found in D1-MSNs and D2-MSNs during the acquisition process, but extinction training selectively decreased the spine density in D1-MSNs. Interestingly, Rac1 was responsible for METH-induced spine plasticity in D1-MSNs but not in D2-MSNs. Additionally, we found that microinjection of a Rac1 inhibitor or activator into the NAc was not sufficient to disrupt reconsolidation, and the pharmacological activation of Rac1 in the NAc facilitated the extinction of METH-associated contextual memory. Regarding cognitive memory, decreased Rac1 activity improved the METH-induced impairment in object recognition memory.

**Conclusion:** Our findings indicate that Rac1 plays opposing roles in the acquisition and extinction of METH-associated contextual memory and reveal the cell-specific role of Rac1 in METH-associated spine remodelling, suggesting that Rac1 is a potential therapeutic target for reducing relapse in METH addiction and remediating METH-induced recognition memory impairment.

## Introduction

Chronic methamphetamine (METH) use leads to impairments in memory (e.g., recognition memory and spatial memory) 1 and the formation of strong, persistent drug-associated memories that significantly contribute to relapse in drug addiction 2. Some effective methods have been developed to prevent cue-induced relapse, including disrupting reconsolidation after retrieval and enhancing extinction 3. Reconsolidation is a labile phase during which retrieved memories may be vulnerable to disruption upon re-exposure to a drug-paired environment. Extinction is generally believed to involve the formation of new inhibitory memories or partial memory erasure by the repeated presentation of paired cues without punishment or reward 3, 4.

Dendritic spines are small, actin-rich, highly motile postsynaptic protrusions from dendritic shafts, and the structural remodelling of spines is associated with many neuropsychiatric and neurodevelopmental disorders 5. Moreover, drug-induced structural plasticity of dendritic spines in the reward circuitry of the brain is crucial for aberrant drug-associated learning and addictive behaviours 6. The nucleus accumbens (NAc) is a key reward centre that plays an important role in drug-associated memory and cognitive memory by receiving dopaminergic projections from the ventral tegmental area and glutamatergic projections from the amygdala, hippocampus and ventral medial prefrontal cortex 7-10. Spine structural plasticity in the NAc is strongly implicated in the acquisition, reconsolidation and extinction of drug-associated memories 11-13. For example, the amphetamine-induced increase in spine density in the NAc is related to drug-associated memory acquisition 14. In addition, latrunculin, which is an actin polymerization inhibitor, reduces the METH-induced increase in dendritic spines and disrupts established METH-associated memories 15. Moreover, the retrieval of a drug-associated memory results in transient increases in the diameter of the dendritic spine head or spine density in the NAc core, while the extinction of a drug-associated memory leads to a decrease in dendritic spine density 13.

Medium spiny neurons (MSNs) are the main neurons in the NAc, and are typically divided into the following two groups: MSNs expressing dopamine receptor type 1 (D1-MSNs) and MSNs expressing dopamine receptor type 2 (D2-MSNs). Accumulative studies have confirmed that D1-MSNs and D2-MSNs in the NAc play different roles in drug-associated memory and display different adaptations in structural and functional synaptic plasticity at different stages of drug-associated memory 16, 17. The activation of D1-MSNs promoted the acquisition of cocaine reward memory, while the activation of D2-MSNs attenuated the acquisition of cocaine reward memory 18. Additionally, previous studies have demonstrated that cocaine-induced spine remodelling occurres in both D1-MSNs and D2-MSNs, but spine remodelling appears to be more stable in D1-MSNs 19. Although the structural changes in dendritic spines have been implicated in different aspects of drug-associated memory, the precise mechanisms by which METH regulates reward-related learning and spine remodelling in each type of MSNs in the NAc remain unknown.

Rac1, a member of the Rho GTPase family of small G proteins, is a crucial mediator of actin dynamics and plays a critical role in regulating spine morphological changes 20-22. Based on accumulating evidence, Rac1 contributes to addiction 23, 24 and diverse forms of learning and memory 5, 25, 26. For instance, excessive Rac1 activity in mice lacking BCR (breakpoint cluster region) or ABR (active BCR-related) impairs cognitive functions such as spatial and object recognition memory 27. In Drosophila, the Rac1-cofilin signal may mediate memory erasure by modulating actin cytoskeleton remodelling 28. Moreover, Rac1 plays a key role in the consolidation of cocaine-associated memory in the NAc and regulates the reconsolidation of cocaine-associated memory in the basolateral amygdala 29.

Considering these findings, in the present study, we hypothesized that Rac1 in the NAc may play an important role in METH-associated contextual memory and spine remodelling. We tested this hypothesis using a conditioned place preference (CPP) model. We demonstrated that reduced Rac1 activity was required for the acquisition of METH-associated contextual memory and spine remodelling in the NAc, while increased Rac1 signalling was important for the extinction of METH-associated contextual memory and the elimination of thin spines. Furthermore, spine remodelling was found in both D1-MSNs and D2-MSNs, and Rac1 activity was associated with spine plasticity in D1-MSNs, but not D2-MSNs, in the NAc. Taken together, our findings highlight the roles of Rac1 in the regulation of METH-associated contextual memory and spine plasticity in D1-MSNs, providing a therapeutic target for the treatment of aberrant addiction memory and improvement of drug-induced memory impairment.

## Methods

### Animals

Adult male C57BL/6J mice aged 6-8 weeks were purchased from the Southern Medical University Animals Centre (Guangzhou, China). Mice weighing 22-25 g were group housed in cages with free access to water and food and subjected to a 12 h light/dark cycle with a constant temperature (22-24 °C). All procedures were performed in accordance with the National Institutes of Health Guide and approved by the Animal Care and Use Committee of the Southern Medical University.

### Drugs

Methamphetamine (METH) was obtained from the Beijing Institute of Pharmacology &Toxicology and dissolved in sterile saline. The Rac1 inhibitor NSC23766 and activator CN04 were purchased from Santa Cruz Biotechnology (Santa Cruz, CA) and Cytoskeleton Inc. (Denver, CO), respectively. NSC23766 was dissolved in sterile saline to 10 µg/μl based on previous studies 29, 30, while CN04 was dissolved in sterile saline to 424 nmol/ml 26. The corresponding sterile saline solution was used as the control treatment. All drugs were freshly prepared.

### Cannula implantation and microinjection

Mice were anaesthetized with pentobarbital sodium (50 mg/kg, i.p.) and placed in a stereotaxic apparatus. Then, mice were bilaterally implanted with 26-gauge guide cannulae (RWD Life Science Co., Ltd.) located 0.5 mm above the NAc at the following coordinates: anteroposterior (AP), +1.54 mm; mediolateral (ML), ±0.80 mm; dorsoventral (DV), -3.90 mm. The cannulae were fixed with dental cement, and a needle was inserted into the cannula to prevent obstruction. These mice were allowed to recover from surgery for 12-14 d before the subsequent behavioural tests. The 28-gauge injection injector protruding 0.5 mm from the guide cannula was connected to Hamilton syringes using polyethylene tubing. NSC23766 (10 µg/μl, 1 µl/side), CN04 (424 nmol/ml, 1 µl/side) or vehicle (1 µl/side) was infused into the bilateral NAc at a rate of 0.25 μl/min. The doses of drugs are based on previous studies 26, 30. After infusion, the injector remained in the cannulae for another 2 min. The correct cannula placement was verified at the end of the experiment using Nissl staining.

### Viral constructs and microinjections

Lentiviral vectors expressing a constitutively active (Rac1-CA) or dominant-negative Rac1 (Rac1-DN) fused with eGFP or lentivirus-eGFP (LV-eGFP) driven by the CMV promoter were constructed as described in our previous study 24. Recombinant adeno-associated serotype 2/9 (AAV 2/9) viruses expressing mCherry driven by the dopamine D1 receptor gene promoter (Drd1-mCherry) or dopamine D2 receptor gene promoter (Drd2-mCherry) were constructed by BrainVTA (Wuhan, China). After placing the anaesthetized mouse in the stereotaxic apparatus, we bilaterally infused the viruses into the NAc over 2 min (coordinates AP, +1.54 mm; ML, ±0.80 mm; and DV, -4.40 mm) using Hamilton syringes connected to a 33-gauge injector. Additionally, the viruses were bilaterally infused in the basolateral amygdala (BLA), hippocampus, or caudate putamen (CPu) according to the following coordinates: BLA (AP, -1.3 mm; ML, ±2.9 mm; and DV, -4.7 mm), hippocampus (AP, -2.1 mm; ML, ±1.3 mm; and DV, -1.9 mm), and CPu (AP, +0.7 mm; ML, ±1.8 mm; and DV, -3.3 mm). The injection needle remained in place for an additional 2 min to allow the virus to diffuse. Two weeks after the injection, which was a sufficient period to allow virus expression and recovery from surgery, the mice were subsequently subjected to behavioural tests.

### Conditioned place preference (CPP) test

Animals were trained using a previously described biased procedure 31. Briefly, the CPP apparatus consisted of three compartments. Two conditioning compartments, separated by a smaller central compartment, were distinguished by floor textures, wall colours and lighting: one had black walls and a smooth floor, whereas the other had white walls, a textured floor, and a light. A camera was placed above the middle of the apparatus to record the time and total distance travelled in each chamber. The CPP procedure consisted of four phases: habituation, baseline preference, conditioning and postconditioning (CPP test 1). On day 1 (habituation), mice were habituated to the testing apparatus with free access to all compartments for 15 min. On day 2 (baseline preference), mice were first placed in the middle chamber and allowed to freely explore the compartments for 15 min; the preferred side was selected as the saline-paired chamber, and the nonpreferred side became the METH-paired chamber. During the conditioning phase (days 3-5), mice were injected with saline and restricted to the preferred side for 30 min in the morning; in the afternoon, mice were injected with saline or METH (2 mg/kg) and confined to the nonpreferred side. On day 6, mice underwent a place preference test identical to the baseline test. The CPP score is expressed as the time spent in the drug-paired chamber minus the time spent in the saline-paired chamber. NAc tissues were dissected immediately after the final METH conditioning training session, and the levels of Rac1 and its downstream targets were investigated.

### Retrieval of METH-associated contextual memory

Mice underwent METH-CPP training to develop associations between one of the CPP chambers and the rewarding effects of METH. Twenty-four h later, mice were re-exposed to the CPP chambers (retrieval) for 15 min to reactivate the METH-associated contextual memory. A significant preference for the METH-paired chamber suggested that the memory has been retrieved. Immediately after memory retrieval, saline or the Rac1 inhibitor (NSC23766; 10 µg/µl; 1 μl/side) was infused bilaterally into the NAc, while the Rac1 activator (CN04; 424 nmol/ml, 1 μl/side) was injected into the NAc 1.5 h before retrieval, because Rac1 reaches maximal activation at approximately 2 h according to previous studies 26. Twenty-four h later, the mice were tested again to evaluate the effect of Rac1 activity on reconsolidation. For western blotting, the mice were decapitated 15 min after memory retrieval.

### Extinction training

The procedure was similar to the METH-induced CPP protocol, with some adjustments based on a previous study 12. During extinction training, mice were confined to the saline-paired or METH-paired chamber in the morning and afternoon, respectively, for 30 min without injections. A 15 min extinction test was performed 24 h after 2 consecutive days of extinction training, including 2 sessions on the METH-paired side and 2 sessions on the saline-paired side. Animals performed six extinction sessions, with each extinction session including two bouts of extinction training and a preference test. The sham extinction group simply underwent six place preference tests without extinction training. For western blotting, the mice were decapitated 30 min after the last extinction training according to previous studies 32. We bilaterally infused the Rac1 activator CN04 into the NAc before extinction training to examine the effects of Rac1 activation on extinction in the CPP.

### Open field test

Mice were placed into the centre of an open field arena (50 cm×50 cm×40 cm) and allowed to freely explore the environment for 10 min. An overhead camera was used to record and assess the animals' basal locomotor activity, as indicated by the total distance moved or the average speed, and anxiety-like behaviour, as measured by the percentages of time spent and distance travelled in the centre of the open field arena (30 cm×30 cm) 33.

### Novel object recognition test (NORT)

The NORT was adapted from methods described in previous reports 27, 33, 34. NORT was performed in an open field apparatus on 3 successive days and included three phases: habituation, training, and testing. In the habituation phase, each animal was placed in a chamber (50 cm×50 cm×40 cm) and allowed to freely explore the arena for 10 min in the absence of objects. On the second day (training phase), the mouse was placed in the same chamber containing two different objects (A and B) and allowed to explore for 5 min. For the testing phase, which occurred 24 h after the sample phase, object B was replaced by a novel object (C), and the time spent exploring objects A and C was recorded when a mouse's nose touched or sniffed the object or when the head of the animal was oriented towards the object and within 2 cm from the object. The exploratory preference index, a ratio of the amount of time spent exploring the novel object (C) to the total amount of time spent exploring both objects, was used to assess memory 34.

### Y-maze

Spatial working memory was evaluated with the spontaneous alternation protocol for the Y-maze described in a previous study 35. Briefly, mice were placed in one arm facing the wall of the arm (the start arm) and allowed to freely explore all three open arms for 5 min, and the numbers of sequential entries into all three arms were recorded with a camera. Sequential entries proceeding clockwise or counter clockwise, such as ACB, CBA, and BAC, were defined as correct, while alternations such as ACA, CAC, and ABA were defined as incorrect. The percentage of spontaneous alternations is the number of correct sequential entries divided by the maximum number of possible alternations (number of total arm entries minus 2) and multiplied by 100. 7 d after the spontaneous alternation test, we also used the Y-maze to test spontaneous spatial novelty preference to assess short-term spatial memory. The procedure was performed using methods described in a previous report 35. Initially, mice were allowed to freely explore two arms of the Y-maze for 5 min, with the remaining arm blocked and defined as the novel arm. After a 15 min interval, mice were allowed to explore all three arms for 5 min unimpeded, and the percentage of time spent in the novel arm was calculated to evaluate short-term spatial memory.

### Dendritic spine analysis of the MSNs

Two weeks after CPP training or extinction training, mice performed a preference test. Twenty-four h later, the animals were sacrificed by transcardial perfusion with PBS and 4% PFA. Brains were postfixed with 4% PFA overnight at 4 °C and then placed in 20% and 30% sucrose solutions for 2 d. Next, brain sections including the NAc were sliced at a thickness of 40 μm using a freezing microtome, and these NAc sections were then immunostained with an anti-GFP antibody (Abcam; 1:500) and an Alexa Fluor 488-conjugated anti-rabbit antibody (Invitrogen; 1:200) as described previously 24. All spine images were captured using a Laser Scanning Confocal Microscope (LSCM, Zeiss LSM 780, Germany). We measured spine density on dendrite sections less than 1.2 μm in width from at least 50 μm of secondary dendrites of eGFP-expressing NAc medium spiny neurons. Spine types were analysed using the semiautomated software Neuron Studio (http://research.mssm.edu/cnic/tools-ns.html). After Neuron Studio processing, an investigator blinded to the experimental conditions verified the spine classification and manually corrected any errors.

### Dendritic spine analysis of the MSNs subtypes

To observe the effects of Rac1 on D1-MSNs and D2-MSNs spine remodelling in the NAc, Drd1-mCherry or Drd2-mCherry were used to specifically label the D1-MSNs or D2-MSNs. The NAc sections were collected from mice infected with Rac1 mutant viruses and either Drd1-mCherry or Drd2-mCherry. These sections were immunostained with an anti-GFP antibody (Abcam; 1:500) and anti-mCherry antibody (Abcam; 1:1000). Then, an Alexa Fluor 488-conjugated anti-rabbit antibody (Invitrogen; 1:200) and Alexa Fluor 594 anti-Chicken (Jackson ImmunoResearch; 1:100) were applied. Only dendritic spines in MSNs labelled with eGFP and mCherry were selected for the spine analysis.

### Rac1 activity assay and western blots analysis

Rac1 activity assay was performed according to our previous study 24. Briefly, Rac1-GTP was pulled down with GST-PAK-PBD agarose, and its activity was examined by western blotting using an anti-Rac1 antibody 22, 24, 36. Western blotting were performed according to our previous papers 24, 37, 38. Primary antibodies for western blotting included the following: anti-Rac1 at a dilution of 1:1000 (BD Transduction Laboratories); 1:1000 p-Pak and 1:1000 Pak (Cell Signaling); 1:1000 p-Limk and 1:1000 Limk (Cell Signaling); 1:1000 p-Cofilin and 1:1000 Cofilin (Cell Signaling); Peroxidase-conjugated goat anti-rabbit or anti-mouse IgG second antibodies were purchased from Santa Cruz Biotechnology Inc. and were used at a dilution of 1:5000.

### Statistical analysis

Statistical analyses were performed using SPSS 13.0 software. Behavioural analyses were performed using a paired t-test, two-way ANOVA or one-way ANOVA followed by Bonferroni's post hoc test. When the two-way ANOVA showed a significant interaction, simple main effects analyses were conducted separately. Western blots were evaluated with Student's t-tests. Data are presented as the mean±SEM. Significance was set at p<0.05.

## Results

### Decreased Rac1 signalling plays an important role in the acquisition of METH-associated contextual memory

**Figure 1:**
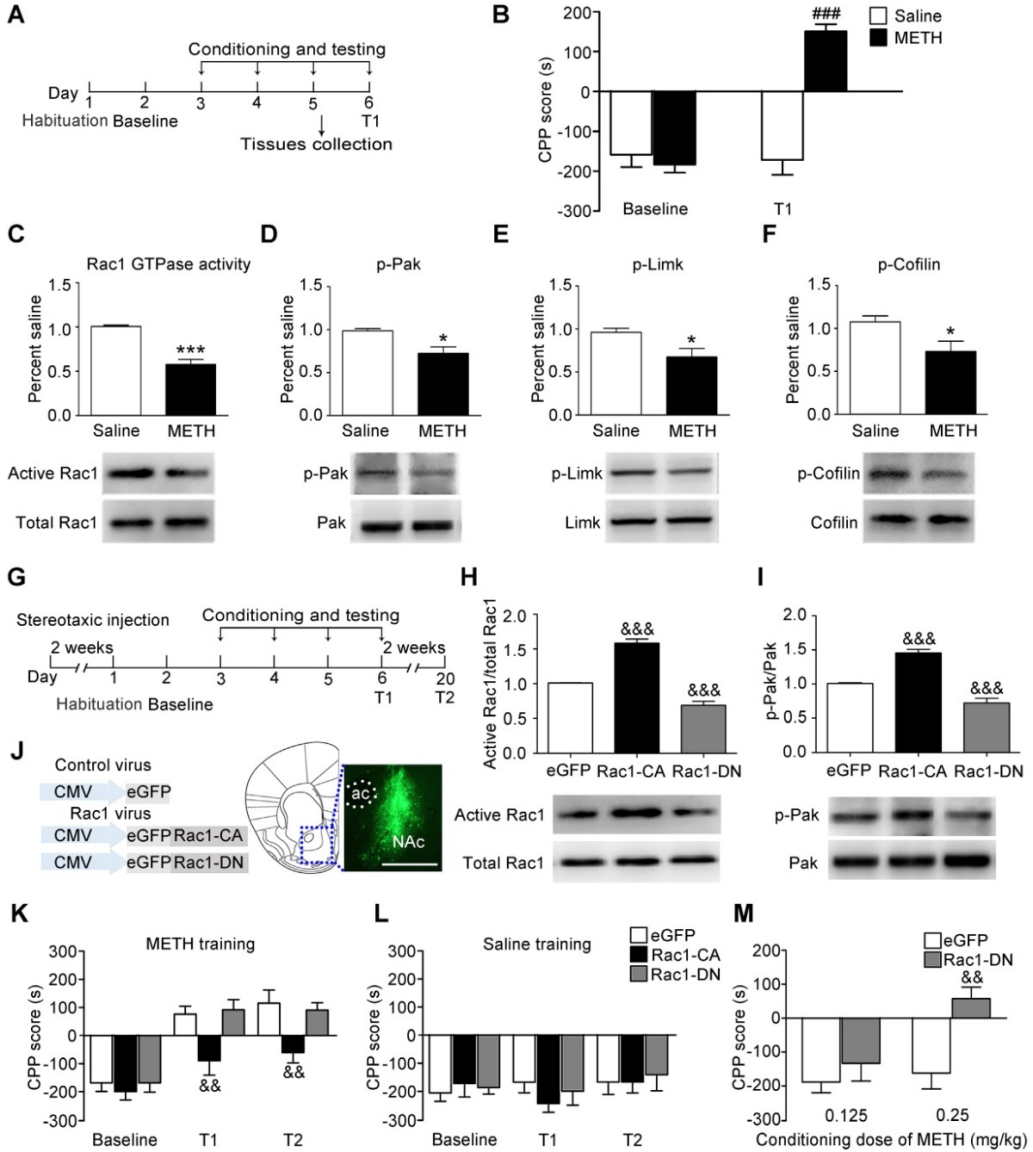
** Decreased Rac1 activity in the NAc is important for METH-associated contextual memory (A)** Experimental paradigm for METH-induced conditioned place preference (CPP). **(B)** Mice conditioned with METH (2 mg/kg, i.p.) exhibited a preference for the METH-paired compartment, while saline training did not result in a significant preference. **(C-F)** Western blots for activated Rac1, p-Pak, p-Limk, and p-Cofilin in the NAc during the acquisition of METH-associated contextual memory.** (G)** Experimental design for METH-induced CPP. **(H-I)** Western blots for Rac1 and p-Pak activity 2 weeks after injection of Rac1 viruses. **(J)** Anatomical location of the NAc in mice injected with a lentivirus expressing eGFP. Scale bar=500 µm. **(K)** Overexpression of Rac1-CA attenuated METH-associated contextual memory in T1 and T2, while Rac1-DN did not further enhance METH-associated contextual memory. **(L)** Rac1 and the control viruses had no effect on the CPP score in saline conditions. **(M)** Overexpression of Rac1-DN promoted METH-CPP acquisition when conditioned with 0.25 mg/kg. Data were analysed using a paired t-test, Student's t-test, two-way or one-way ANOVA followed by Bonferroni's post hoc test and presented as mean±SEM. ###p<0.001, ##p<0.01, and #p<0.05 compared to the baseline preference; ***p<0.001, **p<0.01, and *p<0.05 compared to the saline group; &&&p<0.001, &&p<0.01, and &p<0.05 compared to the eGFP control group. NAc: nucleus accumbens; T1: test 1; T2: test 2.

We used the classical CPP paradigm to establish drug-associated memory. The mice were treated with 2 mg/kg METH or saline for 3 d, and the CPP test was performed 24 h after the final injection of METH (Figure 1A). Under the saline condition, there was no significant difference between baseline and T1 (n=8, t7=0.011, p=0.992, baseline vs. T1, paired t-test, Figure 1B). Under the METH condition, the mice spent more time in the METH-paired compartment than the saline-paired compartment, indicating that 3 d of METH training were sufficient to induce METH-associated contextual memory (n=8, t7=6.930, p<0.001, baseline vs. T1, paired t-test, Figure 1B). Then, we investigated whether Rac1 mediated the acquisition of METH-induced CPP. We found that compared with saline-conditioned mice, the levels of active Rac1 (n=4, t6=7.351, p<0.001, METH vs. saline group, Student's t-test, Figure 1C), p-Pak (n=5, t8=3.325, p=0.010, METH vs. saline group, Student's t-test, Figure 1D), p-Limk (n=5, t8=2.879, p=0.020, METH vs. saline group, Student's t-test, Figure 1E) and phosphorylated Cofilin (n=5, t8=2.430, p=0.041, METH vs. saline group, Student's t-test, Figure 1F) were decreased in METH-conditioned mice. We bilaterally injected Rac1-CA, Rac1-DN or LV-eGFP into the NAc to further assess the role of Rac1 in the acquisition of METH-induced CPP (Figure 1G). Two weeks after the virus injection, Rac1 activity and its downstream p-Pak were assessed by western blotting, which showed that Rac1-CA efficiently increased Rac1 and p-Pak activity, while Rac1-DN decreased Rac1 and p-Pak activity (n=4, Rac1 GTPase activity: F2, 9=139.417, p<0.001; p-Pak : F2, 9=55.54, p<0.001, one-way ANOVA, Bonferroni's post hoc test: Rac1 GTPase activity, p<0.001; p-Pak, p<0.001, Rac1-CA vs. eGFP group; Rac1 GTPase activity, p<0.001; p-Pak, p<0.001, Rac1-DN vs. eGFP group, Figure 1H-I). The immunostaining revealed that the viruses were confined to the NAc (Figure 1J). We found that overexpression of Rac1-CA attenuated the acquisition of METH-associated contextual memory, and this effect persisted for at least 2 weeks (n=8, main effect of the virus: F2, 21=4.958, p=0.017; main effect of the trial: F2, 42=55.68, p<0.001; interaction F4, 42=2.294, p=0.075, two-way ANOVA with repeated measures; Bonferroni's post hoc test: T1, p<0.01; T2, p<0.01, Rac1-CA vs. eGFP group, Figure 1K). Additionally, the intra-NAc injections of Rac1-CA and Rac1-DN had no effect on the natural preference of the mice in the saline condition group (n=8, main effect of virus: F2, 21=1.728, p= 0.065, for trial F2, 42=1.178, p=0.318; interaction F4, 42=0.619, p=0.651, Figure 1L). However, the intra-NAc injection of Rac1-DN did not further strengthen the memory compared with that observed in the control group, potentially due to a ceiling effect because the maximum CPP score was observed at the dose of 2 mg/kg METH in previous studies 39. Thus, we subsequently used a low dose of METH (0.125 mg/kg; 0.25 mg/kg) to evaluate the effect of Rac1-DN on METH-induced CPP according to previous studies 39, 40. We found that Rac1-DN promoted METH-induced CPP following the administration of 0.25 mg/kg METH (n=7, t12=3.791, p=0.003, Rac1-DN vs. eGFP group, Student's t-test, Figure 1M). We further explored whether Rac1 activity was inhibited by METH or the METH-associated memory. We found that Rac1 activity and its downstream targets were decreased 1 h after the final injection without significant alterations 15 min after the final injection (n=4, Rac1 GTPase activity: F2, 9=5.551, p=0.027; p-Pak: F2, 9=18.799, p<0.001, p-Limk: F2, 9=55.602, p<0.001, p-Cofilin: F2, 9=10.638, p=0.004; one-way ANOVA; Bonferroni's post hoc test: Rac1 GTPase activity, p=0.036; p-Pak, p<0.001; p-Limk, p<0.001; p-Cofilin, p=0.012, METH 1 h vs. saline group, Figure S4). Taken together, these data suggest that the METH-induced decrease in Rac1 activity in the NAc could be involved in both the rewarding effects of METH and contextual information processing.

### Decreased Rac1 signalling mediates spine remodelling during the acquisition of METH-associated contextual memory

**Figure 2:**
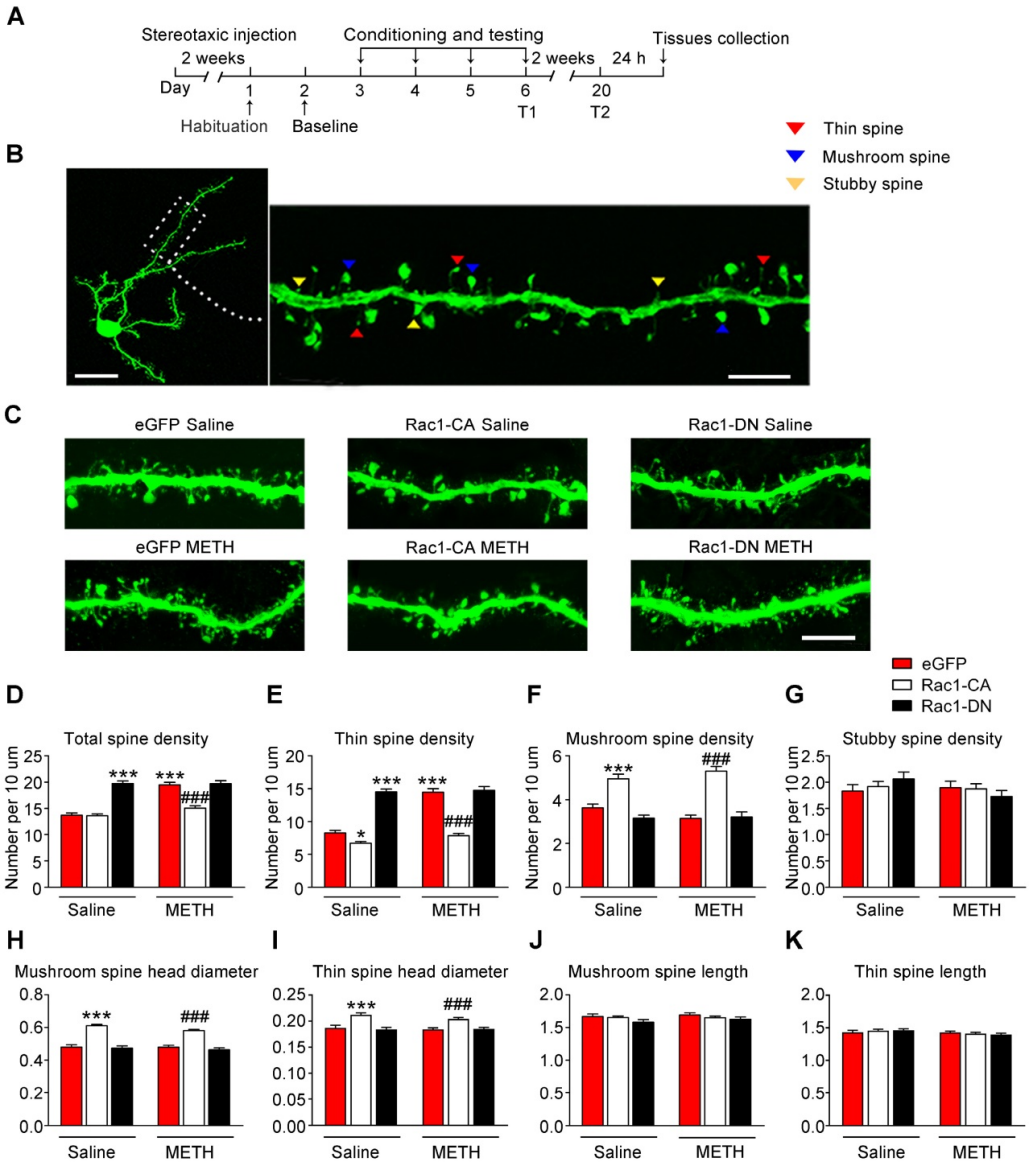
** Decreased Rac1 signalling mediates spine remodelling during the acquisition of METH-associated contextual memory (A)** Experimental design for virus injections and tissues collection. **(B)** Image of a representative eGFP-labelled NAc medium spiny neuron with an adjacent dendritic segment showing three types of spines. Scale bar for the left image=20 µm and for the right image=5 µm. **(C)** Representative confocal images of dendritic segments in mice injected with different viruses expressing eGFP after treatment with saline or METH. Scale bar=5 µm. **(D)** METH significantly increased the total spine density in the NAc. Overexpression of Rac1-CA reversed the METH-induced increase in total spine density, while overexpression of Rac1-DN plus the saline treatment increased the total spine density compared to eGFP saline controls. **(E)** Repeated METH treatments increased the density of thin dendritic spines. A post hoc Bonferroni analysis revealed a significant increase in the thin spine density of the Rac1-DN saline group, while the Rac1-CA saline group showed a decreased thin spine density compared with eGFP saline controls. Additionally, eGFP METH-treated mice showed an increase in the thin spine density that was reversed by the overexpression of Rac1-CA. **(F)** The METH treatment had no effect on the mushroom spine density, while both the Rac1-CA saline and Rac1-CA METH groups showed an increased number of mushroom spines. **(G)** Neither the METH treatments nor the Rac1 virus altered the stubby spine density on NAc medium spiny neurons. **(H)** Under both saline and METH conditions, overexpression of Rac1-CA increased the mushroom head diameter. **(I)** Overexpression of Rac1-CA increased the thin spine head diameter under both saline and METH conditions. **(J-K)** No significant differences in the mushroom and thin spine length were observed between all groups. Data were analysed using two-way ANOVA followed by Bonferroni's post hoc test and presented as mean±SEM. ***p<0.001, **p<0.01 and *p<0.05 compared to the eGFP saline group; ###p<0.001, ##p<0.01, and #p<0.05 compared to the eGFP METH group. T1: test 1; T2: test 2.

According to numerous studies, structural plasticity in the NAc is an essential neurobiological substrate of reward-cue memory 14. We bilaterally infused Rac1-CA, Rac1-DN, or the LV-eGFP control vector into the NAc to examine the role of Rac1 in the plasticity of spines in NAc medium spiny neurons. After 2 weeks, the mice expressing these viruses were subjected to METH or saline CPP training. Twenty-four h after CPP test 2, we prepared brain slices to analyse spine remodelling in the MSNs (Figure 2A). The following three distinct subtypes of spines were quantified in the MSNs: thin spines, stubby spines, and mushroom spines (Figure 2B). We found that METH treatment increased the total spine density (8-10 dendrite sections per animal with 4-5 animals per group, main effect of the group: F1, 198=40.844, p<0.001; main effect of the virus: F2, 198=68.600, p<0.001; interactions: F2, 198=21.162, p<0.001, two-way ANOVA; Bonferroni's post hoc test: p<0.001, eGFP METH vs. eGFP saline group, Figure 2D), and that this increase was largely due to an increase in the density of the thin spines (main effect of the group: F1, 198=50.537, p<0.001; main effect of the virus: F2, 198=144.912, p<0.001; interactions: F2, 198=26.894, p<0.001, two-way ANOVA; Bonferroni's post hoc test: p<0.001, eGFP METH vs. eGFP saline group, Figure 2E), without significant changes in the mushroom (main effect of METH: F1, 198=0.035, p=0.851; main effect of the virus: F2, 198=64.958, p<0.001; interactions: F2, 198=2.500, p=0.085, two-way ANOVA, Figure 2F) or stubby spine density (main effect of the group: F1, 198=1.050, p=0.307; main effect of the virus: F2, 198=0.406, p=0.667, interactions: F2, 198=1.274, p=0.282, two-way ANOVA, Figure 2G). A post hoc Bonferroni analysis revealed that overexpression of Rac1-CA significantly decreased the total spine density (p<0.001, Rac1-CA METH vs. eGFP METH group) and thin spine density (p<0.001, Rac1-CA METH vs. eGFP METH group) induced by METH CPP training (Figure 2D-E), while the overexpression of Rac1-DN alone increased the thin spine density (p<0.001, Rac1-DN saline vs. eGFP saline group), which is similar to the effect of METH on spine plasticity (Figure 2E). Thus, a decrease in Rac1 activity is necessary for the METH-induced changes in spine density in the NAc. Additionally, we analysed the head diameter and length of the mushroom and thin spines. We found that the METH treatment had no effect on the mushroom spine head diameter (main effect of the group: F1, 1977=2.277, p=0.131, main effect of the virus: F2, 1977=113.132, p<0.001, interactions: F2, 1977=1.229, p=0.293, two-way ANOVA, Figure 2H), thin spine head diameter (main effect of the group: F1, 3796=1.279, p=0.258, main effect of the virus: F2, 3976=25.060, p<0.001, interactions: F2, 3976=0.698, p=0.498, two-way ANOVA, Figure 2I), length of the mushroom spines (main effect of the group: F1, 1977=0.751, p=0.386, main effect of the virus: F2, 1977=2.042, p=0.130, interactions: F2, 1977=0.344, p=0.709, two-way ANOVA, Figure 2J) and length of the thin spines (main effect of the group: F1, 3796=3.510, p=0.061, main effect of the virus: F2, 3976=0.021, p=0.979, interactions: F2, 3976=0.911, p=0.402, two-way ANOVA, Figure 2K), but Rac1-CA overexpression alone increased the mushroom spine head diameter (p<0.001, Rac1-CA saline vs. eGFP saline group) and thin spine head diameter (p<0.001, Rac1-CA saline vs. eGFP saline group) without affecting the spine length (Figure 2H-K), suggesting that Rac1 plays an important role in spine head morphology.

### Effect of Rac1 signalling on the reconsolidation of METH-associated contextual memory

**Figure 3:**
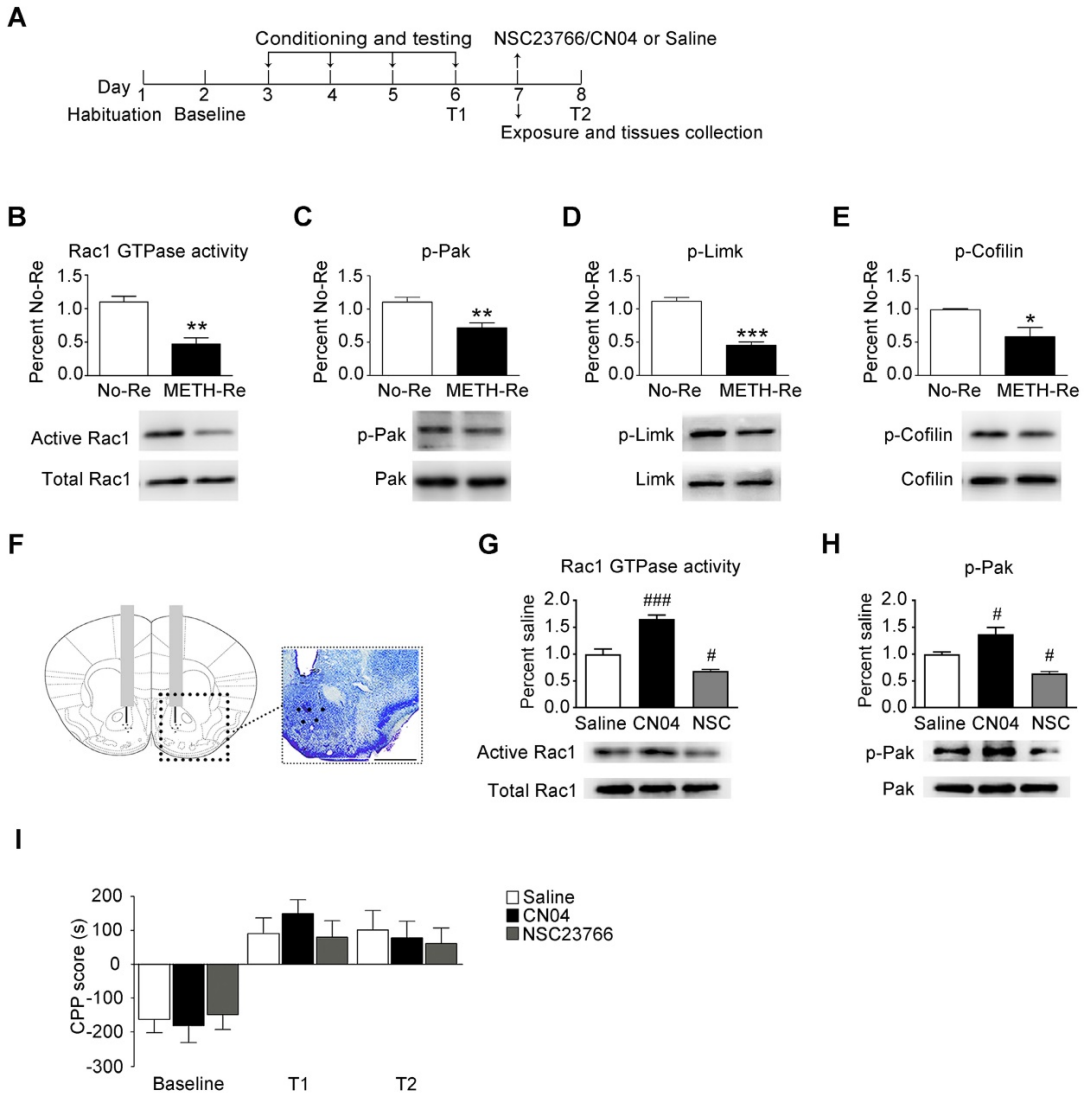
** Effect of Rac1 signalling on the reconsolidation of METH-associated contextual memory (A)** Timeline of the METH-CPP training and microinjections. **(B-E)** Western blots showing the levels of activated Rac1 and its downstream targets 15 min after the retrieval of METH-associated contextual memory. **(F)** Representative images of cannula placements in the NAc. Scale bar=500 µm. **(G-H)** Western blots of Rac1 and p-Pak activity after the injection of CN04 and NSC23766. **(I)** The microinjection of NSC23766 or CN04 into the NAc had no effect on the reconsolidation of METH-associated contextual memory. Data were analysed using Student's t-test or two-way ANOVA followed by Bonferroni's post hoc test and presented as mean±SEM. *p<0.05, **p<0.01, and ***p<0.001 compared to the No-Retrieval group. ###p<0.001, ##p<0.01, and #p<0.05 compared to the saline group. METH-Re: METH-Retrieval; No-Re: No-Retrieval; NSC: NSC23766; T1: test 1; T2: test 2.

When drug-associated contextual memory is reactivated, the memory is thought to be in a destabilized state and becomes susceptible to disruption. Subsequently, we assessed whether Rac1 signalling was involved in the reconsolidation of METH-associated contextual memory. The mice were conditioned as described above. After the METH-associated memory formation, the mice were divided into 2 groups. One group was re-exposed to the METH-paired compartment to reactivate the METH-cued memories, and then, the NAc tissues were collected 15 min after retrieval. The other group was decapitated at the same time without retrieval (Figure 3A). We found that Rac1 activity and the levels of p-Pak, p-Limk and phosphorylated Cofilin were decreased (n=4, Rac1 GTPase activity: t6=5.004, p=0.002; p-Pak: t6=3.764, p=0.009; p-Limk: t6=8.794, p<0.001; p-Cofilin: t6=2.936, p=0.026, METH retrieval vs. no retrieval group, Student's t-test, Figure 3B-E). Then, we injected saline, a Rac1 inhibitor (NSC23766) or a Rac1 activator (CN04) into the NAc to examine the role of Rac1 in reconsolidation. The effect of NSC23766 and CN04 on Rac1 activity was examined by western blotting. Because previous studies suggest that CN04 activates Rac1 activity robustly at approximately 2 h 41, we collected NAc tissues 2 h after the administration of NSC23766 or CN04. We found that the Rac1 inhibitor (NSC23766) efficiently decreased Rac1 and p-Pak activity, while the Rac1 activator (CN04) increased Rac1 and p-Pak activity (n=4, Rac1 GTPase activity: F2, 9=48.142, p<0.001; p-Pak: F2, 9=21.209, p<0.001; one-way ANOVA; Bonferroni's post hoc test: Rac1 GTPase activity, p<0.001; p-Pak, p=0.027, CN04 vs. saline group; Rac1 GTPase activity, p=0.039; p-Pak, p=0.032, NSC23766 vs. saline group, Figure 3G-H). These results indicate that NSC23766 and CN04 are capable of regulating Rac1 activity in the NAc. Moreover, no differences were observed among all groups after Rac1 inhibition or activation during reconsolidation (n=7, main effect of the treatment: F2, 18=0.163, p=0.851; main effect of the trial: F2, 36=26.589, p<0.001; interaction: F4, 36=0.360, p=0.836, Figure 3I), indicating that reconsolidation is independent of the decrease in Rac1 activity.

### Increased Rac1 signalling plays an important role in the extinction of METH-associated contextual memory

**Figure 4:**
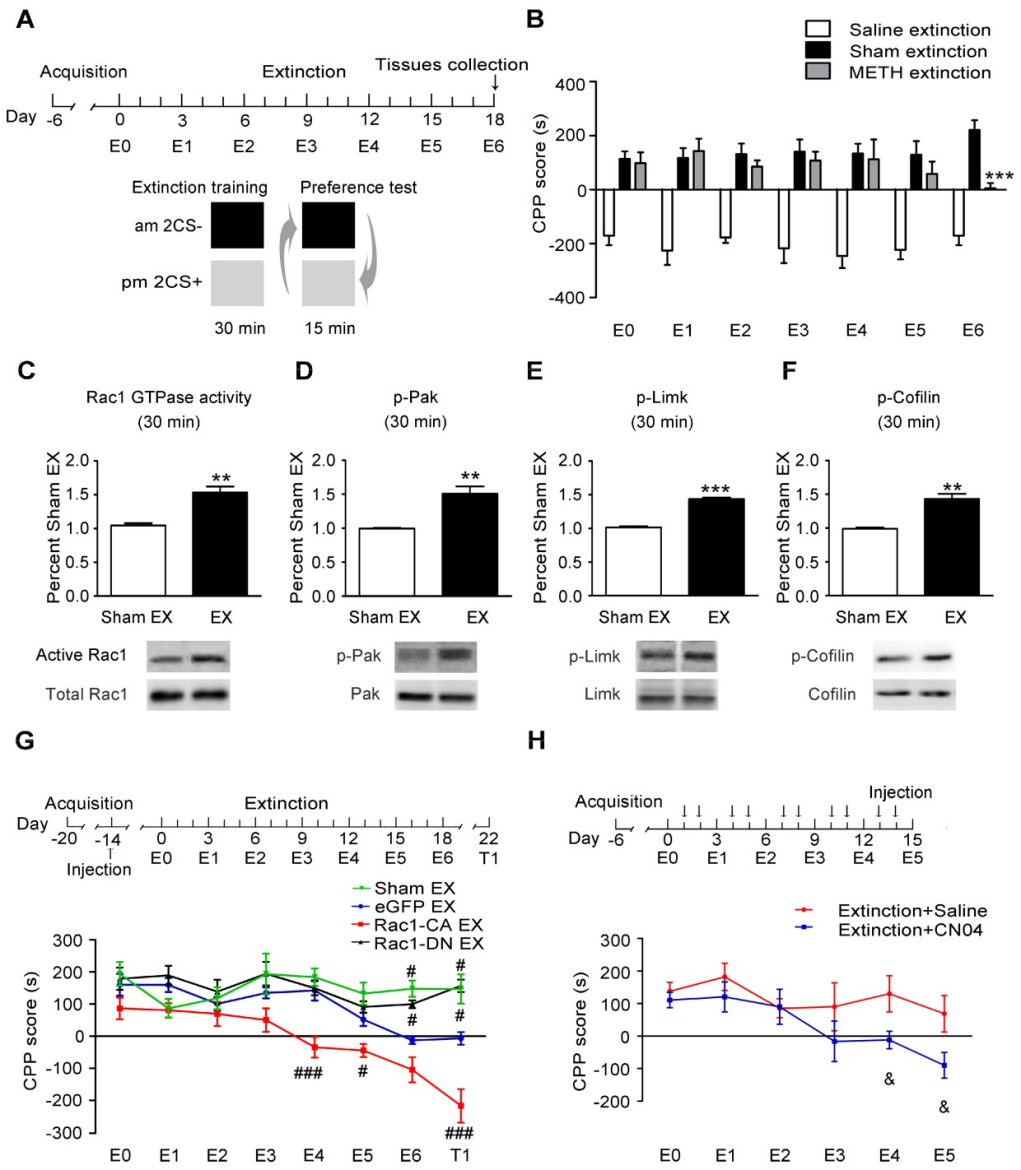
** Increased Rac1 activity facilitates the extinction of METH-associated contextual memory (A)** Design of the extinction training experiment. **(B)** Extinction training induced a significant decrease in the preference for the METH-paired compartment on E6. **(C-F)** Western blots showing the levels of activated Rac1 and its downstream targets during METH extinction training. **(G)** Effects of Rac1 mutant viruses on the extinction of METH-CPP. Mice expressing Rac1-CA showed a reduced CPP score on E4, E5 and T1, while overexpression of Rac1-DN blocked the extinction of METH-associated contextual memory on E6 and T1. **(H)** Mice were subjected to the extinction procedure and the Rac1 activator (CN04; 424 nmol/ml, 1 μl/side) was injected into the NAc 1 h before extinction training. CN04-injected mice showed a reduced preference for the METH-paired compartment on E4 and E5 compared with the saline-injected mice. Data were analysed using Student's t-test or two-way ANOVA followed by Bonferroni's post hoc test and presented as mean±SEM. ***p<0.001, **p<0.01, and *p<0.05 compared to the sham extinction group; ###p<0.001, ##p<0.01, and #p<0.05 compared to the eGFP extinction groups; &&&p<0.001, &&p<0.01, and &p<0.05 compared to the extinction+saline group. CS+: the METH-paired compartment; CS-: the saline-paired compartment; Ex: extinction; T1: test 1.

The mice were trained to establish METH-associated contextual memory as described above and then underwent extinction training to investigate the potential role of Rac1 in the extinction of METH-induced CPP (Figure 4A). We found that the extinction training significantly decreased the animals' preference for the METH-paired compartment on E6 (n=7-8, main effect of the group: F2, 18=121.561, p<0.001; two-way ANOVA with repeated measures; Bonferroni's post hoc test: p<0.001, METH extinction vs. sham extinction group, Figure 4B), indicating that established METH-associated contextual memory was partially eliminated by the repeated presentation of a drug-paired context. Compared with the sham extinction group, the western blotting revealed a significant increase in the levels of activated Rac1 30 min after the extinction training (n=4, t6=5.453, p=0.002, extinction vs. sham extinction group, Student's t-test, Figure 4C). The extinction training also resulted in the activation of Pak1 and Limk1 and the inactivation of Cofilin (n=4, p-Pak: t6=4.821, p=0.002; p-Limk: t6=15.53, p<0.001; p-Cofilin: t6=5.745, p=0.001, extinction vs. sham extinction group, Student's t-test, Figure 4D-F). Based on these results, Rac1 signalling may play an important role in extinction training. The mice that received the Rac1-CA, Rac1-DN or LV-eGFP injection were divided into the following four groups to test this hypothesis: three extinction groups, including the Rac1-CA extinction group, the Rac1-DN extinction group and the LV-eGFP extinction group, which were subjected to daily extinction training, and a control group expressing eGFP that did not undergo extinction training. According to the Bonferroni's post hoc test, overexpression of Rac1-CA in the NAc promoted the extinction of the METH reward memory on E4 (p<0.001, Rac1-CA extinction vs. eGFP extinction group) and E5 (p<0.05, Rac1-CA extinction vs. eGFP extinction group), while overexpression of Rac1-DN impaired the extinction of METH-associated contextual memory on E6 (p=0.017, Rac1-DN extinction vs. eGFP extinction group) and T1 (p=0.02, Rac1-DN extinction vs. eGFP extinction group) compared with the eGFP extinction group (n=8-10, main effect of the group: F3, 31=28.315, p<0.001; main effect of the trial: F7, 217=11.829, p<0.001; interaction: F21, 217=3.416, p<0.001, two-way ANOVA with repeated measures, Figure 4G), indicating that Rac1 activity was required for the extinction of METH-associated contextual memory. Furthermore, the mice injected with Rac1-CA still showed a significantly reduced preference for the METH-paired compartment two weeks after the extinction training (p<0.001, Rac1-CA extinction vs. eGFP extinction group, Figure 4G), indicating that Rac1 exerts long-lasting effect on the promotion of the extinction of drug reward memory. Subsequently, we investigated the effects of the Rac1 activator CN04 on extinction training. CN04 or saline was bilaterally microinjected into the NAc 1 h before extinction training. The mice injected with CN04 showed a reduced preference for the METH-paired compartment in trials E4-5 compared with the mice in the saline control group (n=7, main effect of the group: F1, 12=6.416, p=0.026; main effect of the trial: F5, 60=3.383, p=0.009; interaction: F5, 60=0.963, p=0.448, two-way ANOVA with repeated measures; Bonferroni's post hoc test: p<0.05, extinction CN04 vs. extinction saline group, Figure 4H). These results confirm the findings obtained using Rac1-CA.

### Role of Rac1 signalling in spine remodelling during the extinction of METH-associated contextual memory

**Figure 5:**
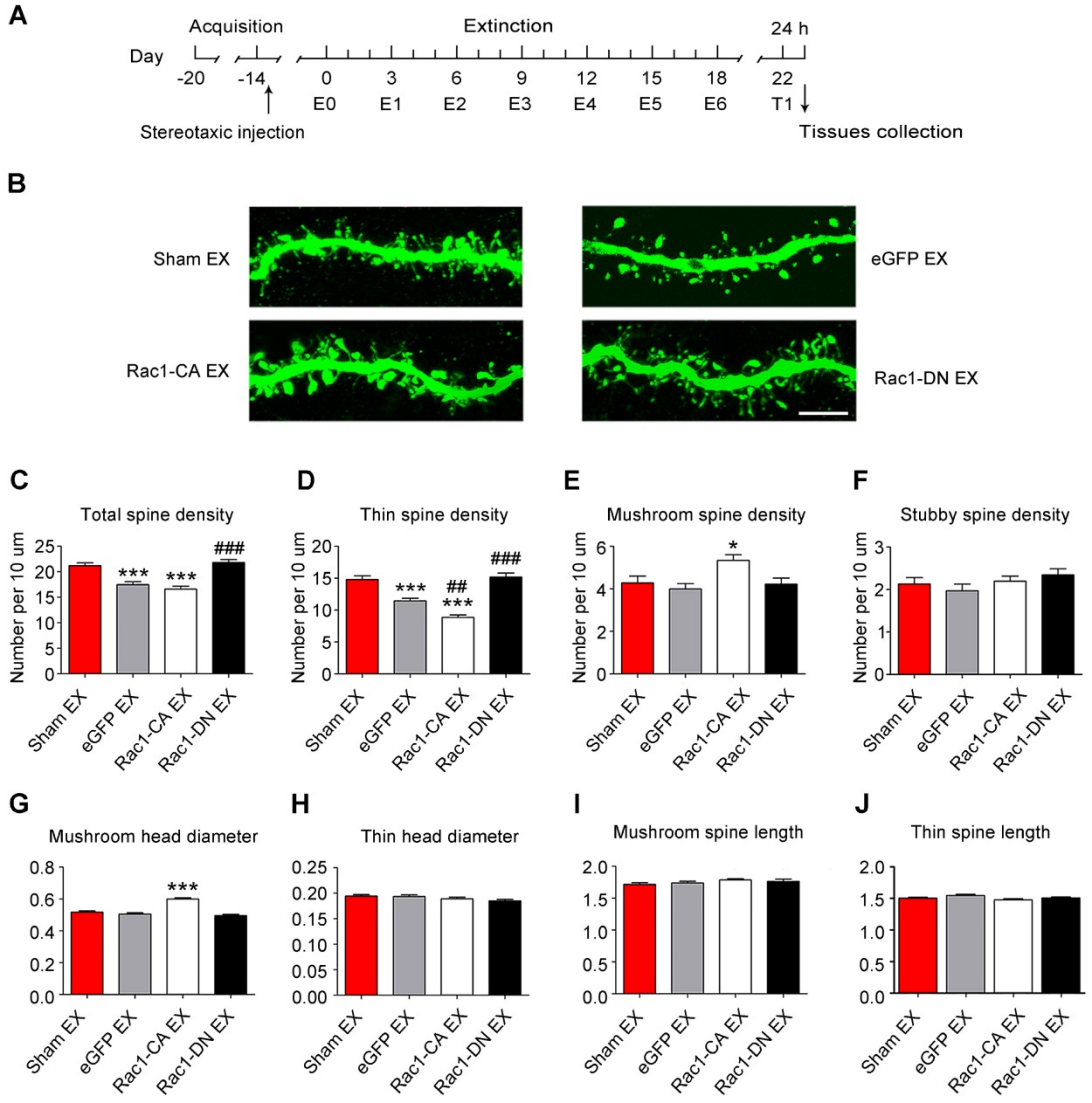
** Increased Rac1 signalling mediates spine remodelling during the extinction of METH-associated contextual memory (A)** Experimental design for the extinction procedure and tissues collection.** (B)** Representative confocal images of dendritic segments. Scale bar=5 µm. **(C)** Extinction training induced a significant decrease in total spine density, while Rac1-DN overexpression blocked this effect. **(D)** Extinction training resulted in a decrease in the thin spine density. Overexpression of Rac1-DN blocked the extinction-induced reduction in the thin spine density, while overexpression of Rac1-CA promoted the elimination of thin spines compared with the eGFP extinction group. **(E)** The eGFP injection and extinction training had no effect on the mushroom spine density, while overexpression of Rac1-CA increased the number of mushroom spines.** (F)** No difference in stubby spine density was observed between all groups. **(G)** The eGFP injection and extinction training had no effect on the mushroom head diameter, while the overexpression of Rac1-CA induced a significant increase in mushroom head diameter compared to the sham extinction group. **(H-J)** No differences in thin spine head diameter, mushroom spine length and thin spine length were observed between all groups. Data were analysed using one-way ANOVA followed by Bonferroni's post hoc test and presented as mean±SEM. ***p<0.001, **p<0.01, and *p<0.05 compared with the sham extinction groups; ###p<0.001, ##p<0.01, and #p<0.05 compared with eGFP extinction control groups. EX: extinction; T1: test1.

Given that the METH-CPP conditioning training induced the formation of thin spines in the NAc, which is believed to be the structural basis of METH-associated contextual memory, we further explored whether METH-induced CPP extinction affected dendritic spine remodelling and whether Rac1 participated in this remodelling process. Following the METH conditioning training, Rac1-CA, Rac1-DN or LV-eGFP was injected into the mouse NAc, and then, the animals were subjected to six extinction sessions. Two weeks after the extinction training, a CPP test was performed to determine whether the extinguished memory persisted, and then, the dendritic spines in the NAc were examined 24 h after the CPP test (Figure 5A). The extinction training reduced the total spine density compared with that in the sham extinction group (5-7 dendritic segments per mouse and 5-6 mice per group, F3, 138=20.934, p<0.001, one-way ANOVA; Bonferroni's post hoc test: p<0.001, eGFP extinction vs. sham extinction group, Figure 5C), and the reduction in spine density was mainly due to the decrease in the thin spine density (F3, 138=39.41, p<0.001, one-way ANOVA; Bonferroni's post hoc test: p<0.001, eGFP extinction vs. sham extinction group, Figure 5D). Additionally, the Rac1-CA overexpression accelerated the elimination of thin spines (p<0.01, Rac1-CA extinction vs.eGFP extinction group), while the Rac1-DN overexpression blocked the reduction in the thin spine density (p<0.001, Rac1-DN extinction vs. eGFP extinction group, Figure 5D). Thus, Rac1 activity is essential for extinction-induced spine remodelling. In addition, the overexpression of Rac1-CA increased the mushroom spine density (F3, 138=5.280, p=0.002, one-way ANOVA, Bonferroni's post hoc test: p=0.038, Rac1-CA extinction vs. sham extinction group, Figure 5E) and mushroom head diameter (F3, 1792=39.481, p<0.001, one-way ANOVA, Bonferroni's post hoc test: p<0.001, Rac1-CA extinction vs. sham extinction, Figure 5G), suggesting that Rac1-CA may selectively eliminate immature thin spines and increase the mushroom spine density and head diameter.

### Effects of Rac1 activity on METH-induced cognitive impairment

**Figure 6:**
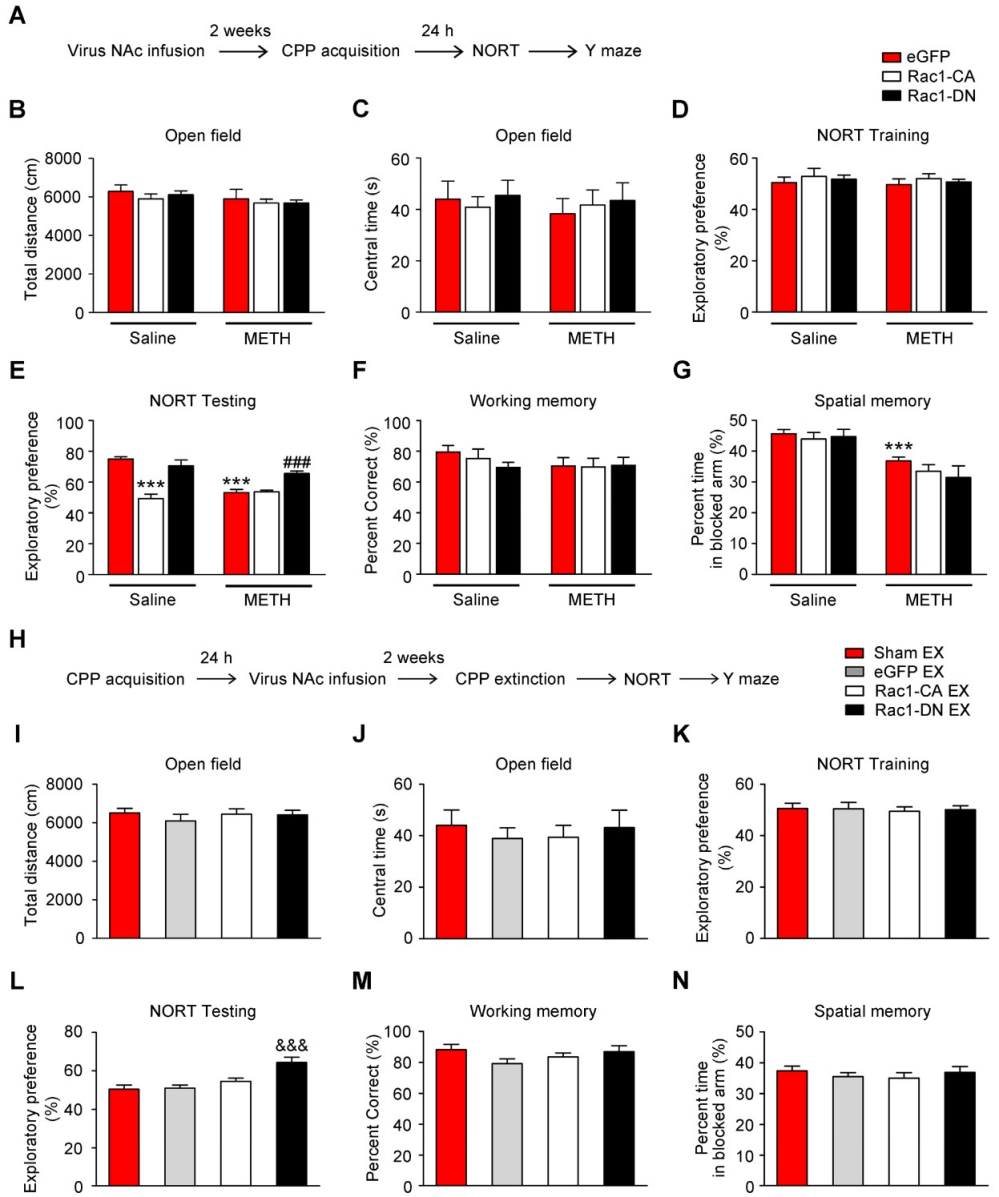
** Effects of Rac1 activity and extinction training on METH-induced cognitive impairment (A)** Diagram of the experimental procedure.** (B-C)** No differences in the total distance travelled and time spent in the centre were observed between all groups. **(D)** During the NORT training phase, differences in exploratory preference were not observed between all conditions. **(E)** During the NORT testing phase, repeated METH treatment reduced the exploratory preference. Under the saline conditions, overexpression of Rac1-CA reduced the exploratory preference compared to the eGFP saline group, while overexpression of Rac1-DN increased the exploratory preference compared to the eGFP METH group under METH conditions. **(F)** The mean percentage of correct spontaneous alternations was not different between all groups. **(G)** Repeated METH treatment reduced the percent time spent exploring the previously blocked arm. **(H)** Diagram of the experimental procedure.** (I)** Comparison of the total distances travelled by all groups. **(J)** Comparison of the centre times of all groups. **(K)** Significant differences in the level of exploratory preference for the novel objects were not observed during the training phase. **(L)** Overexpression of Rac1-DN increased the exploratory preference. **(M-N)** Significant differences in the mean percentage of correct spontaneous alternations and percent time spent exploring the previously blocked arm were not observed. Data were analysed using two-way or one-way ANOVA followed by Bonferroni's post hoc test and presented as mean±SEM. ***p<0.001, **p<0.01, and *p<0.05 compared to the eGFP saline group; ###p<0.001, ##p<0.01, and #p<0.05 compared to the eGFP METH group; &&&p<0.001, &&p<0.01, and &p<0.05 compared to the sham extinction group. CPP: conditioned place preference; EX: extinction; NORT: the novel object recognition test.

Numerous studies have illustrated the important roles of Rac1 in different types of memory, such as object recognition memory and spatial memory 27, 33, 42. Additionally, emerging evidence indicates the involvement of the NAc in object recognition memory and spatial memory, in addition to its roles in reward and addiction 43, 44. However, little is known about the effect of Rac1 expressed in the NAc on METH-induced memory impairments. Therefore, the mice were injected with Rac1-CA, Rac1-DN or LV-eGFP and subjected to saline or METH conditioning training and memory tests, including NORT, spatial working memory test and short-term spatial memory test (Figure 6A). One d after the saline- or METH-induced CPP training, the NORT was performed. The METH-CPP training had no effect on the total distance travelled (n=8, main effect of the group: F1, 42=2.127, p=0.152, two-way ANOVA, Figure 6B) or time spent in the centre (n=8, main effect of the group: F1, 42=0.217, p=0.644, two-way ANOVA, Figure 6C) compared to the saline-treated mice, suggesting that locomotor activity and anxiety were not affected by the METH-CPP training. During the NORT training session, no significant difference in exploratory preference was observed among the groups (n=8, main effect of the group: F1, 42=9.706, p=0.603, two-way ANOVA, Figure 6D), indicating that these mice did not have an inherent preference for each object. However, during the NORT testing session, the METH-treated group showed a significantly decreased exploratory preference for the novel objects compared to the saline-treated group, and this decrease was improved by Rac1-DN (n=8, main effect of the group: F1, 42=15.563, p<0.001; main effect of the virus: F1, 42=27.697, p<0.001; interaction: F1, 42=16.393, p<0.001, two-way ANOVA; Bonferroni's post hoc test: p<0.001, eGFP METH vs. eGFP saline group; p<0.001, Rac1-DN METH vs eGFP METH group, Figure 6E).

Similar to the METH exposure, overexpression of Rac1-CA plus saline CPP training induced a significant decrease in the exploratory preference for the novel objects (p<0.001, Rac1-CA saline vs. eGFP saline group, Figure 6E). Based on these results, Rac1 activation is linked to METH-induced memory impairment in novel object recognition. Subsequently, the mice were placed in a Y-maze to evaluate their spatial working memory and short-term spatial memory as previously reported 35. We found that the METH-treated mice showed no significant difference in the percentage of correct alternating arm entries (n=7, main effect of the group: F1, 36=1.372, p=0.249, main effect of the virus: F2, 36=0.543, p=0.585; interaction: F2, 36=0.677, p=0.514, two-way ANOVA, Figure 6F), but spent less time in the novel arm than the saline-treated mice (n=7, main effect of the group: F1, 36=32.99, p<0.001; main effect of the virus: F2, 36=1.046, p=0.3619; interaction: F2, 36=0.4732, p=0.6268, two-way ANOVA; Bonferroni's post hoc test: p<0.001, eGFP METH vs. eGFP saline group, Figure 6G), suggesting that METH-induced CPP induces deficits in short-term spatial memory without affecting spatial working memory. Additionally, as shown in Figure 6F-G, Rac1-CA, Rac1-DN, and LV-eGFP had no effect on the METH-induced short-term spatial memory impairments. Furthermore, we explored the effect of extinction training on the METH-induced cognitive impairment. Mice that had previously established a METH-induced CPP were injected with Rac1-CA, Rac1-DN or LV-eGFP and subjected to the behavioural tests described above (Figure 6H). We found that the eGFP extinction group did not exhibit significant differences in their performance in the open field test (n=8-10, total distance: F3, 33=0.463, p=0.709; central time: F3, 33=0.235, p=0.870, one-way ANOVA, Figure 6 I-J), the NORT (n=8-10, F3, 33=9.526, p<0.001, one-way ANOVA; Bonferroni's post hoc test: p=1.000, eGFP extinction vs. sham extinction group, Figure 6L) and the short-term spatial memory test (n=8-9, F3, 31=0.444, p=0.723, one-way ANOVA, Figure 6N) from the eGFP sham extinction group, suggesting that the extinction training has no effect on the METH-induced memory impairment. Consistent with the results described above, overexpression of Rac1-DN after METH treatments improved the METH-induced impairment in object recognition memory (n=8-10, F3, 33=9.526, p<0.001, one-way ANOVA; Bonferroni's post hoc test: p<0.001, Rac1-DN extinction vs. sham extinction group, Figure 6L) without affecting working memory (n=8-9, F3, 31=1.537, p=0.225 one-way ANOVA, Figure 6M) and short-term spatial memory (n=8-9, F3, 31=0.444, p=0.723, one-way ANOVA, Figure 6N), supporting the protective effect of decreased Rac1 activity on impairments in object recognition memory.

### Cell-type and region-specific role of Rac1 signalling during the METH-associated contextual memory

**Figure 7:**
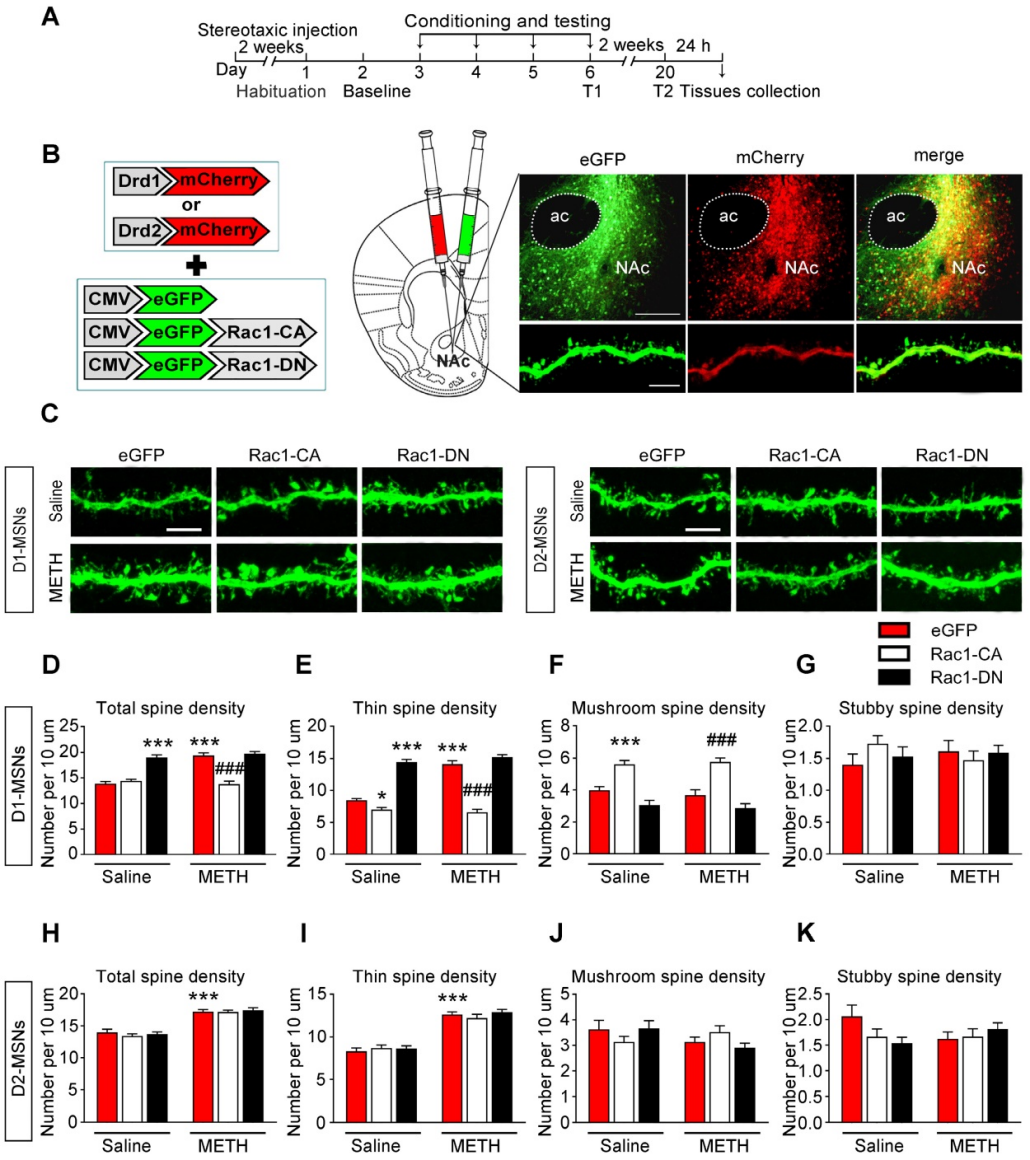
** Decreased Rac1 signalling mediates spine remodelling in D1-MSNs during the acquisition of METH-associated contextual memory (A)** Experimental design for the acquisition of procedure and tissues collection.** (B)** Injection schematic. Rac1 mutant viruses and either Drd1-mCherry or Drd2-mCherry were bilaterally injected into the NAc and only dendritic spines in MSNs labelled with eGFP and mCherry were selected for the spine analysis. Representative image of the NAc and a dendrite infected with both mCherry and eGFP. Scale bar=200 µm (upper) and 5 µm (below). **(C)** Representative confocal images of dendritic segments in D1-MSNs and D2-MSNs in each group. Scale bar=5 µm. **(D-E)** METH significantly increased the total and thin spine density in D1-MSNs, which was reversed by Rac1-CA. Rac1-CA saline groups showed a decreased thin spine density.The total and thin spine density in D1-MSNs were increased by Rac1-DN under the saline conditions. **(F-G)** METH treatment had no effect on the mushroom and stubby spine density in D1-MSNs. Rac1-CA increased the mushroom spine density under the saline and METH conditions. **(H-K)** METH significantly increased the total and thin spine density in D2-MSNs. Rac1-CA and Rac1-DN had no effect on the spine density in D2-MSNs. Data were analysed using two-way ANOVA followed by Bonferroni's post hoc test and presented as mean±SEM. ***p<0.001, **p<0.01 and *p<0.05 compared to the eGFP saline group; ###p<0.001, ##p<0.01, and #p<0.05 compared to the eGFP METH group. D1-MSNs: medium spiny neurons expresing dopamine receptor type 1; D2-MSNs: medium spiny neurons expressing dopamine receptor type 2; NAc: nucleus accumbens; T1: test 1; T2: test 2.

MSNs are predominantly divided into two groups that express either the D1 or D2 dopamine receptor. D1-MSNs and D2-MSNs play opposing roles in regulating reward-related behaviour and display different structural plasticity during drug addiction 19, 45. However, little is known of cell-type-specific spine plasticity induced by METH. To further explore whether different changes occur in spine remodelling between D1-MSNs and D2-MSNs in the NAc and dentify the type of MSNs that is regulated by Rac1, the mice were injected with Rac1 mutant viruses and either Drd1-mCherry or Drd2-mCherry, and only dendritic spines from MSNs labelled with both eGFP and mCherry were selected for the spine analysis (Figure 7A-B). An increase in total spine density was found in both D1-MSNs (8-10 dendritic segments per mouse and 3 mice per group, main effect of the group: F1, 178=24.827, p<0.001; main effect of the virus: F2, 178=66.969, p<0.001; interactions: F2, 178=24.395, p<0.001, two-way ANOVA; Bonferroni's post hoc test: p<0.001, eGFP METH vs. eGFP saline group, Figure 7D) and D2-MSNs (8-10 dendritic segments per mouse and 3 mice per group, main effect of the group: F1, 178=144.689, p<0.001; main effect of the virus: F2, 178=0.434, p=0.648; interactions: F2, 178=0.263, p=0.769, p<0.001, two-way ANOVA; Bonferroni's post hoc test: p<0.001, eGFP METH vs. eGFP saline group, Figure 7H) during the acquisition of METH-CPP, and METH mainly increased the thin spine density (D1-MSNs: main effect of the group: F1, 178=38.688, p<0.001; main effect of the virus: F2, 178=210.284, p<0.001; interactions: F2, 178=32.705, p<0.001; D2-MSNs: main effect of the group: F1, 178=192.199, p<0.001; main effect of the virus: F2, 178=0.497, p=0.609; interactions: F2, 178=0.797, p=0.452, two-way ANOVA; Bonferroni's post hoc test: p<0.001, eGFP METH group vs. eGFP saline group, Figure 7E and I) without affecting mushroom (D1-MSNs: main effect of the group: F1, 178=0.264, p=0.608; main effect of the virus: F2, 178=52.682, p<0.001; interactions: F2, 178=0.386, p=0.680; D2-MSNs: main effect of the group: F1, 178=1.883, p=0.172; main effect of the virus: F2, 178=0.065, p=0.937; interactions: F2, 178=2.836, p=0.061, two-way ANOVA, Figure 7F and J) and stubby spine density (D1-MSNs: main effect of the group: F1, 178=0.001, p=0.972; main effect of the virus: F2, 178=0.209, p=0.811; interactions: F2, 178=1.348, p=0.262; D2-MSNs: main effect of the group: F1, 178=0.176, p=0.675; main effect of the virus: F2, 178=0.813, p=0.445; interactions: F2, 178=2.744, p=0.067, two-way ANOVA, Figure 7G and K) on both MSNs cell types. Additionally, Rac1-CA significantly reversed METH-induced increase in the total spine density (p<0.001, Rac1-CA METH vs. eGFP METH group) and thin spine density (p<0.001, Rac1-CA METH vs. eGFP METH group) in D1 MSNs (Figure 7D and E), but not in D2-MSNs (Figure 7H and I), while Rac1-DN alone selectively increased the thin spine density in D1 MSNs (p<0.001, Rac1-DN saline vs. eGFP saline group, Figure 7E). Thus, the decreased Rac1 signalling is necessary for METH-induced spine remodelling in D1-MSNs but not D2-MSNs during the acquisition of METH-associated contextual memory.

**Figure 8:**
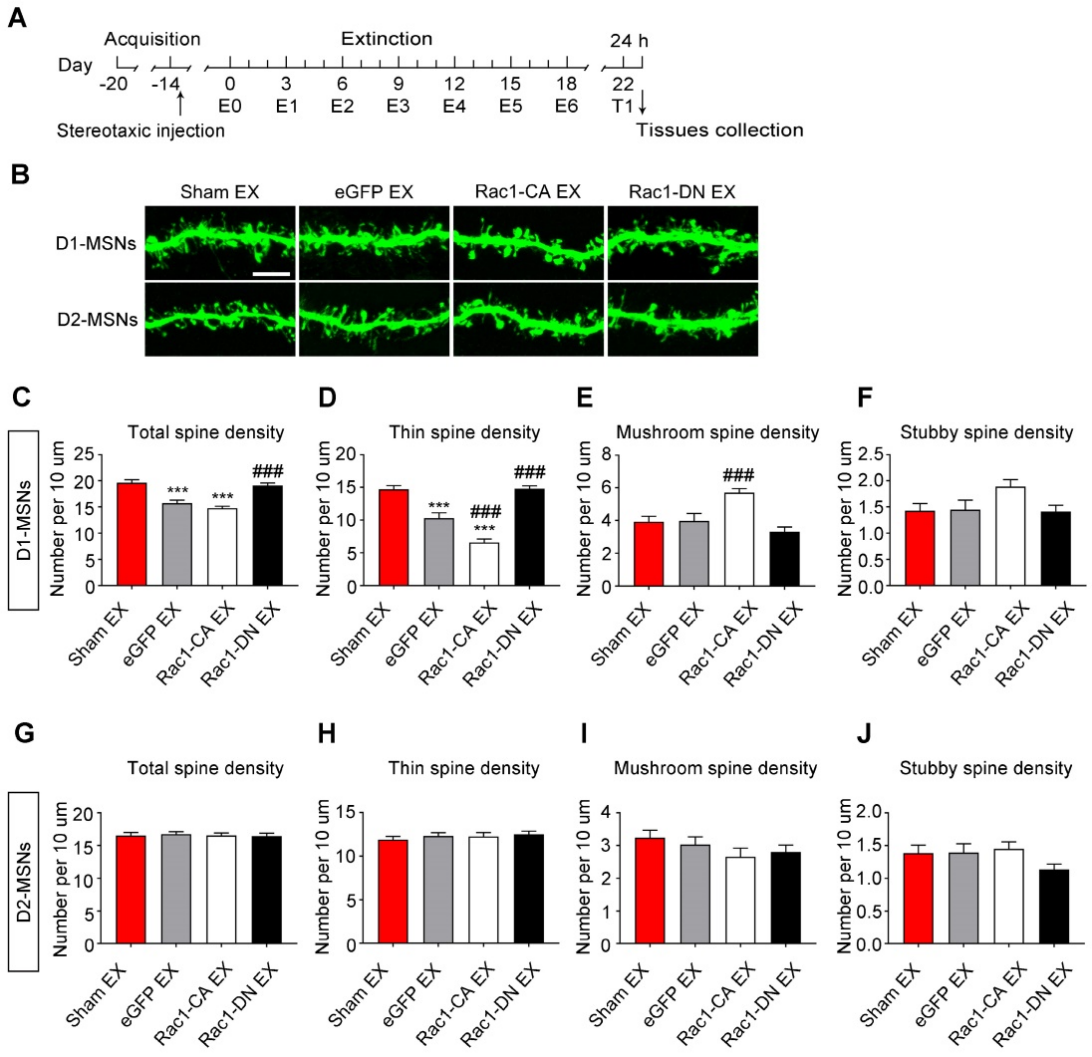
** Increased Rac1 signalling mediates spine remodelling in D1-MSNs during the extinction of METH-associated contextual memory (A)** Experimental design for the extinction procedure and tissues collection. **(B)** Representative confocal images of dendritic segments in D1-MSNs and D2-MSNs. Scale bar=5 µm.** (C-D)** Extinction training resulted in a decrease in the total and thin spine density in D1-MSNs. Rac1-DN blocked the extinction-induced reduction in the thin spine density, while Rac1-CA promoted the elimination of thin spines in D1-MSNs. **(E-F)** Rac1-CA increased the mushroom spine density without affecting the stubby spine density in D1-MSNs. **(G-J)** Comparison of spine density between all groups. Extinction training had no effect on spine density in D2-MSNs. Data were analysed using one-way ANOVA followed by Bonferroni's post hoc test and presented as mean±SEM. ***p<0.001, **p<0.01, and *p<0.05 compared with the sham extinction groups; ###p<0.001, ##p<0.01, and #p<0.05 compared with eGFP extinction control groups. D1-MSNs: medium spiny neurons expresing dopamine receptor type 1; D2-MSNs: medium spiny neurons expressing dopamine receptor type 2; EX: extinction; T1: test 1.

In addition, we found that the extinction training selectively decreased the total (8-10 dendritic segments per mouse and 3 mice per group, F3, 127=24.492, p<0.001, one-way ANOVA; Bonferroni's post hoc test: p<0.001, eGFP extinction vs. sham extinction group, Figure 8C) and thin spine density (F3, 127=48.579, p<0.001, one-way ANOVA; Bonferroni's post hoc test: p<0.001, eGFP extinction vs. sham extinction group, Figure 8D) in D1-MSNs, and Rac1-CA promoted the elimination of thin spines in D1-MSNs (p<0.001, Rac1-CA extinction vs. eGFP extinction group, Figure 8D), while Rac1-DN had the opposite effects (p<0.001, Rac1-DN extinction vs. eGFP extinction group, Figure 8D). Furthermore, the extinction training had no effect on the spine density in D2-MSNs (8-10 dendritic segments per mouse and 3 mice per group, total spine density: F3, 130=0.097, p=0.962; thin spine density: F3, 130=0.404, p=0.750; mushroom spine density: F3, 130=1.122, p=0.343; stubby spine density: F3, 130=1.625, p=0.187; one-way ANOVA, Figure 8G-J). These results indicate that increased Rac1 activity is associated with spine plasticity in D1-MSNs but not D2-MSNs during the extinction of METH-associated contextual memory.

Taken together, our results indiacate that Rac1 plays a cell-specific role in METH-associated spine remodelling in the NAc. Subsequently, we further assessed whether the involvement of Rac1 in METH-associated contextual memory was only specific to the NAc. We found that METH decreased Rac1 activity and p-Pak activity in the CPu (n=4-5, Rac1 GTPase activity: t7=4.481, p<0.01; p-Pak: t7=5.275, p<0.01, Student's t-test, METH vs. saline group, Figure S1B-C), but increased Rac1 and p-Pak activity in the hippocampus (n=4, Rac1 GTPase activity: t6=-3.827, p=0.009; p-Pak: t6=-2.640, p=0.039; Student's t-test, METH vs. saline group, Figure S1F-G) and BLA (n=4, Rac1 GTPase activity: t6=-3.069, p=0.022; p-Pak: t6=-3.404, p=0.014, p<0.001; Student's t-test, METH vs. saline group, Figure S1J-K). Additionally, we found that both increasing hippocampus Rac1 activity (n=7-8, F2, 19=22.18, p<0.001, one-way ANOVA; Bonferroni's post hoc test: p<0.001, Rac1-DN METH vs. eGFP METH group, Figure S1I) and decreasing CPu Rac1 activity (n=7-8, F2, 19=18.99, p<0.001, one-way ANOVA; Bonferroni's post hoc test: p<0.001, Rac1-CA METH vs. eGFP METH group, Figure S1E) were essential for METH-induced CPP. However, the inhibiton of Rac1 in the BLA had no effect on METH-induced CPP (n=7-8, F2, 19=9.365, p<0.01, one-way ANOVA; Bonferroni's post hoc test: p>0.05, Rac1-DN METH vs. eGFP METH group, Figure S1M). We also examined the effect of Rac1 signalling on METH-induced spine remodelling in the CPu, hippocampus, and BLA. As shown in Figure S2, Rac1-CA attenuated METH-induced increases in total spine density (8-10 dendritic segments per mouse and 3 mice per group, F2, 88=147.211, p<0.001, one-way ANOVA; Bonferroni's post hoc test: p<0.001, Rac1-CA METH vs. eGFP METH group, Figure S2C) and thin spine density in the CPu (8-10 dendritic segments per mouse and 3 mice per group, F2, 88=173.078, p<0.001, one-way ANOVA; Bonferroni's post hoc test: p<0.001, Rac1-CA METH vs. eGFP METH group, Figure S2D), whereas Rac1-DN led to a decrease in the total spine density (8-10 dendritic segments per mouse and 3 mice per group, F2, 88=73.974, p<0.001, one-way ANOVA; Bonferroni's post hoc test: p<0.001, Rac1-DN group vs. eGFP METH group, Figure S2G) and thin spine density (8-10 dendritic segments per mouse and 3 mice per group, F2, 88=106.878, p<0.001, one-way ANOVA; Bonferroni's post hoc test: p<0.001, Rac1-DN METH vs. eGFP METH group, Figure S2H) induced by METH in the hippocampus but not BLA (8-10 dendritic segments per mouse and 3 mice per group, total spine density: F2, 84=28.319, p<0.001; thin spine density: F2, 84=30.648, p<0.001; one-way ANOVA, Bonferroni's post hoc test: p>0.05, Rac1-DN METH vs. eGFP METH group, Figure S2K-L). These results indicate that Rac1 plays a region-specific role in METH-associated contextual memory and spine remodelling.

## Discussion

Drug addiction is a chronic disease, and numerous attempts have been made in the treatment of addiction 46. Drug-associated memory significantly contributes to the relapse of drug-seeking behaviour; thus, an understanding of the molecular mechanism underlying this unwanted and pathological memory process may have important implications for the treatment of addiction 3. Our study highlights the important roles of Rac1 in different METH-associated contextual memory processes and cognitive deficits, and reveals a molecular mechanism by which Rac1 regulates activity-induced spine remodelling in METH addiction, indicating that Rac1 is a potent therapeutic target for the manipulation of METH addiction behaviours.

NAc, a well-known reward centre, has been implicated in the cravings associated with drug addiction 47, and spine morphological changes in the NAc have been observed in a METH behavioural sensitization model 48. Rac1, a major regulator of the actin cytoskeleton, is heavily implicated in spine plasticity, which is thought to be the primary mechanism underlying learning and memory 20. Rac1 may regulate dendritic spine plasticity and memory decay via the actin-severing factor Cofilin, an intermediate in the Pak pathway 28. In the present study, Rac1 signalling in the NAc was essential for METH-associated contextual memory acquisition and thin spine formation. These results are consistent with previous studies showing that decreased Rac1 signalling is essential for cocaine-induced addition memory and structural plasticity in the NAc 23, 24. Furthermore, our observation of reduced Cofilin phosphorylation is consistent with the study conducted by Shibasaki et al., who showed that ADF/Cofilin plays an important role in the development of methamphetamine-induced place preference 49. The excess activation of Rac1 results in selective shrinkage and reduced numbers of immature spines 50, 51. Consistent with these findings, we found that overexpression of Rac1-CA significantly decreased the thin spine density and reversed the METH-induced increase in thin spines. Additionally, the analysis of the spine head diameter and spine length revealed that overexpression of Rac1-CA increased the mushroom head diameter, which is consistent with a previous report showing that Rac1 plays an important role in spine head morphology 52.

Based on evidence from rodent models, Rac1 expressed in the CA1 is required for the reconsolidation of contextual fear memory 53, while Rac1 expressed in the basolateral amygdala is critical for the reconsolidation of cocaine-associated memory 29. In the present study, we found that the retrieval of METH-contextual memories decreased Rac1 activity, which was accompanied by a reduction in the levels of p-Pak, p-Limk and phosphorylated Cofilin. However, the pharmacological activation or inhibition of Rac1 had no effect on disrupting METH-associated contextual memory. Therefore, either the Rac1 pathway is not involved in the reconsolidation of METH-associated contextual memory or Rac1 activation by CN04 is not sufficient to disrupt all reconsolidation processes, including protein synthesis, neurotransmitter receptor-mediated neurotransmission and AMPA trafficking 3, 54.

Extinction is defined as a lessening of the response to conditioned cues following the repeated presentation of paired cues, and numerous studies suggest that extinction involves the formation of new inhibitory memories 3. In contrast, emerging evidence also indicates that partial memory erasure occurs during extinction 4. It has been reported that METH extinction training decreases the synaptic AMPA/NMDA ratio, resulting in the partial erasure of METH-associated contextual memory, and an actin cycling inhibitor has been shown to disrupt METH-associated memories and spine remodelling in the basolateral amygdala 15, 31. Whether the old memory is suppressed or erased following extinction is still controversial. It is possible that the new memory acquisition and old memory erasure coexist during the extinction training 55. Recent studies investigating fear learning suggest that the inhibition mechanism is predominant during the early phase of extinction training, whereas the original fear memory is erased after repeated extinction training 56. Consistent with the view of the extinction of fear memory, it has been shown that extended cue extinction reversed the synaptic changes induced by cocaine self-administration, whereas brief cue extinction had no effect on synaptic plasticity 57. In our study, we found that multiple extinction sessions resulted in the activation of Rac1 signalling and a decrease in the thin spine density in the NAc. Additionally, the overexpression of Rac1-CA reversed the METH-induced increase in the thin spine density and promoted the extinction of a METH-associated contextual memory. Our data are consistent with a previous study showing that Rac1 activation is required for the extinction of aversive memories of drug withdrawal 32. The increase in spine density observed in the NAc has been postulated to be associated with the formation of drug-associated memory 14. Thus, considering the multiple extinction sessions used in our study and the decreased thin spine density observed in the NAc after the extinction training, the effect of Rac1 on METH extinction is likely to be at least partially associated with the erasure of the METH-associated contextual memory.

Under nonpathological conditions, Rac1 is tightly correlated with dendritic spine morphogenesis and long-term potentiation (LTP)-like synaptic modifications underlying memory storage 20. In our study, Rac1 activation was required for the extinction of METH-associated contextual memory and the elimination of thin spines. These apparently contradictory findings may be due to the examination of different memory stages or different disease models in which Rac1 activity may regulate spine dynamics through different mechanisms, thus exerting distinct effects on spine formation and shrinkage or elimination. Indeed, many studies have shown the involvement of Rac1 in memory forgetting and extinction, in addition to memory storage. For instance, the inhibition of Rac1 activity in the hippocampus impairs the extinction of contextual fear memory 26 and interference-induced forgetting of object recognition memory 33. Moreover, Rac1 activation at synapses is required for the internalization of AMPARs and spine shrinkage during long-term depression (LTD) 58, and elevated Rac1 activity reverses previous LTP and even causes significant LTD induced by paired pulse low-frequency stimulation 33. Additionally, Rac1 activity is required for NMDAR-mediated LTD, which is an indispensable mechanism underlying fear extinction 59, 60. Therefore, the effect of Rac1 on METH extinction is likely mediated by LTD induction and spine elimination, thus resulting in the partial erasure of the METH-associated contextual memory. In addition, the findings of a previous study using AS-PaRac1 (Activated Synapse targeting Photoactivatable Rac1) to elicit the shrinkage and elimination of recently potentiated spines showed motor memory erasure 61, further supporting the role of Rac1 in spine elimination and memory erasure.

Accumulating evidence indicates that repeated METH administration impairs object recognition and spatial memory, and that these memory impairments can persist for at least 28 d after drug withdrawal 34, 62. The NAc, which acts through glutamatergic and dopaminergic transmission or through cross-talk with the PFC and hippocampus, plays an important role in object recognition memory and spatial memory 43, 44, 63. To date, only a few studies have investigated the role of the NAc in drug-induced cognitive dysfunction 62. In the present study, the METH conditioning training impaired long-term object recognition memory and short-term spatial memory without affecting spontaneous alternation behaviours in a Y-maze task, which is consistent with previous studies investigating the effects of METH on cognition 34, 64. Additionally, researchers have proposed that repeated METH administration may selectively impair the long-term retention of object recognition memory (24 h test), rather than dampening recognition memory acquisition or short-term recognition memory retention 34. Interestingly, we found that overexpression of Rac1-CA alone impaired object recognition memory, and that the decreased Rac1 activity improved METH-induced object recognition memory impairment. It has been reported that repeated METH treatment reduces basal dopamine concentrations in the NAc during early withdrawal 65, 66, and reduced dopamine levels in the NAc are associated object memory impairment (24 h test) 44. It is also shown that dopamine receptor expressed in mushroom bodies (Damb) could mediate forgetting through Rac1 pathway at low levels of dopamine 67-69. Thus it is possible that METH-induced reduction in dopamine concentrations in the NAc during early withdrawal may impair object memory impairment through activation of Rac1. In further support, others have found that excess Rac1 activity impairs object recognition memory (24 h test) and reduces LTP maintenance 27, and that the inhibition of Rac1 activity leads to a slower object recognition memory forgetting 28, 33. Therefore, we speculate that decreased Rac1 activity may protect the long-term retention of object recognition memory to a certain extent, thereby improving the METH-induced object recognition memory impairments. We also explored whether extinction training affects object recognition and spatial memory. Our results demonstrate that METH-induced persistent cognitive deficits are not associated with the extinction of METH-associated contextual memory.

The major cell populations (90-95%) of the NAc neurons are MSNs, which are typically divided into the following two groups on the basis of their expression of dopamine receptors: D1-MSNs and D2-MSNs. It has been reported that D1-MSNs and D2-MSNs display differential structural adaptations and molecular changes, resulting in opposing behavioural outcomes 16, 70. For instance, the deletion of TrkB in D1-MSNs increased cocaine CPP, while the deletion of TrkB in D2-MSNs had the opposite effect on cocaine reward 18. RhoA is involved in regulation of depression-like behaviours via dendritic remodelling in NAc D1-MSNs but not D2-MSNs 71. Therefore, we further explored the type of MSNs that was regulated by Rac1 during METH-associated contextual memory. We found that the spine density in both D1-MSNs and D2-MSNs was significantly increased during acquisition of METH-CPP. Additionally, Rac1-CA significantly decreased the total spine density and thin spine density in D1-MSNs, but not D2-MSNs, while Rac1-DN alone selectively increased the thin spine density in D1-MSNs. In addition, we found that the extinction training selectively decreased the thin spine density in D1-MSNs, and that Rac1-CA accelerated the elimination of thin spines. Our results indicate that Rac1 was mainly responsible for METH-induced spine plasticity in D1-MSNs. A recent study demonstrated that conditional knockout of WAVE1 in D1-MSNs but not D2-MSNs attenuated cocaine-induced CPP 72. Morover, another study revealed that the activation of Rap1 in D1-MSNs contributed to cocaine-induced CPP 73. Taken together, these results highlight the predominant role of D1-MSNs rather than D2-MSNs in drug reward memory. Importantly, our data are in support of the previous findings that D1-MSNs but not D2-MSNs contributes to the cocaine-mediated decrease of Tiam1, an upstream regulator of Rac1, in the NAc 74. In addition, other studies showd that cocaine-induced CPP was linked to synaptic potentiation between the hippocampus and NAc 75, and silencing excitatory hippocampal input onto NAc D1-MSNs impaired contextual reward behavior 76. We found that the extinction training selectively decreased the thin spine density in D1-MSNs. One possibility is that NAc D1-MSNs receive more inputs from the hippocampus encoding spatial and contextual information 77. We speculate that the decrease in spine density in D1-MSNs may represent a decreased excitatory synaptic contact between the hippocampus and NAc which contributes to the extinction of METH-associated contextual memory.

## Conclusions

In summary, Rac1 in the NAc exerts opposing effects on the acquisition and extinction of METH-associated contextual memory and spine remodelling, and is mainly responsible for the METH-induced spine plasticity in D1-MSNs. To the best of our knowledge, this study is the first to show that Rac1 activity plays different roles in the acquisition and extinction of METH-associated contextual memory, and suggest that Rac1 activity is associated with spine plasticity in D1-MSNs, but not D2-MSNs, in the NAc. Moreover, our study highlights not only the role of Rac1 in drug reward memory but also the role of the NAc in drug-induced cognitive impairments. However, the precise intracellular mechanisms underlying the effects of Rac1 on METH-associated contextual memory and cognitive impairments remain to be elucidated. Taken together, our findings provide a promising therapeutic target for the reduction of cue-induced relapse in METH addiction and a new opportunity to combat the cognitive deficits induced by METH exposure.

## Abbreviations

AMPA: α-amino-3-hydroxy-5-methyl-4-isoxazolepropionic acid receptor
BLA: basolateral amygdala
CPP: conditioned place preference
CPu: caudate putamen
D1-MSNs: medium spiny neurons expresing dopamine receptor type 1
D2-MSNs: medium spiny neurons expressing dopamine receptor type 2
Damb: dopamine receptor expressed in mushroom bodies
EX: extinction
Hip: hippocampus
LTP: long-term potentiation
LTD: long-term depression
NAc: nucleus accumbens
METH: methamphetamine
METH-Re: METH-Retrieval
MSNs: medium spiny neurons
No-Re: no-retrieval
NAc: nucleus accumbens
NMDA: n-methyl-d-aspartate
NORT: novel object recognition test
PBS: phosphate buffer saline
PFA: paraformaldehyde
Rac1: ras-related C3 botulinum toxin substrate 1
Tiam1: t-lymphoma invasion and metastasis 1
TrkB: tropomyosin related kinase receptor subtype B
WAVE1: wiskott-Aldrich syndrome protein family verprolin homologous protein
