# Supplementary material for: Different roles of Rac1 in the acquisition and extinction of methamphetamine-associated contextual memory in the nucleus accumbens
Source: Theranostics. 2019 Sep 21;9(23):7051–71. doi: 10.7150/thno.34655 (PMC6815963; doi:10.7150/thno.34655)
Supplement: Supplementary file 1 — Supplementary figures and tables. [file thnov09p7051s1.pdf]

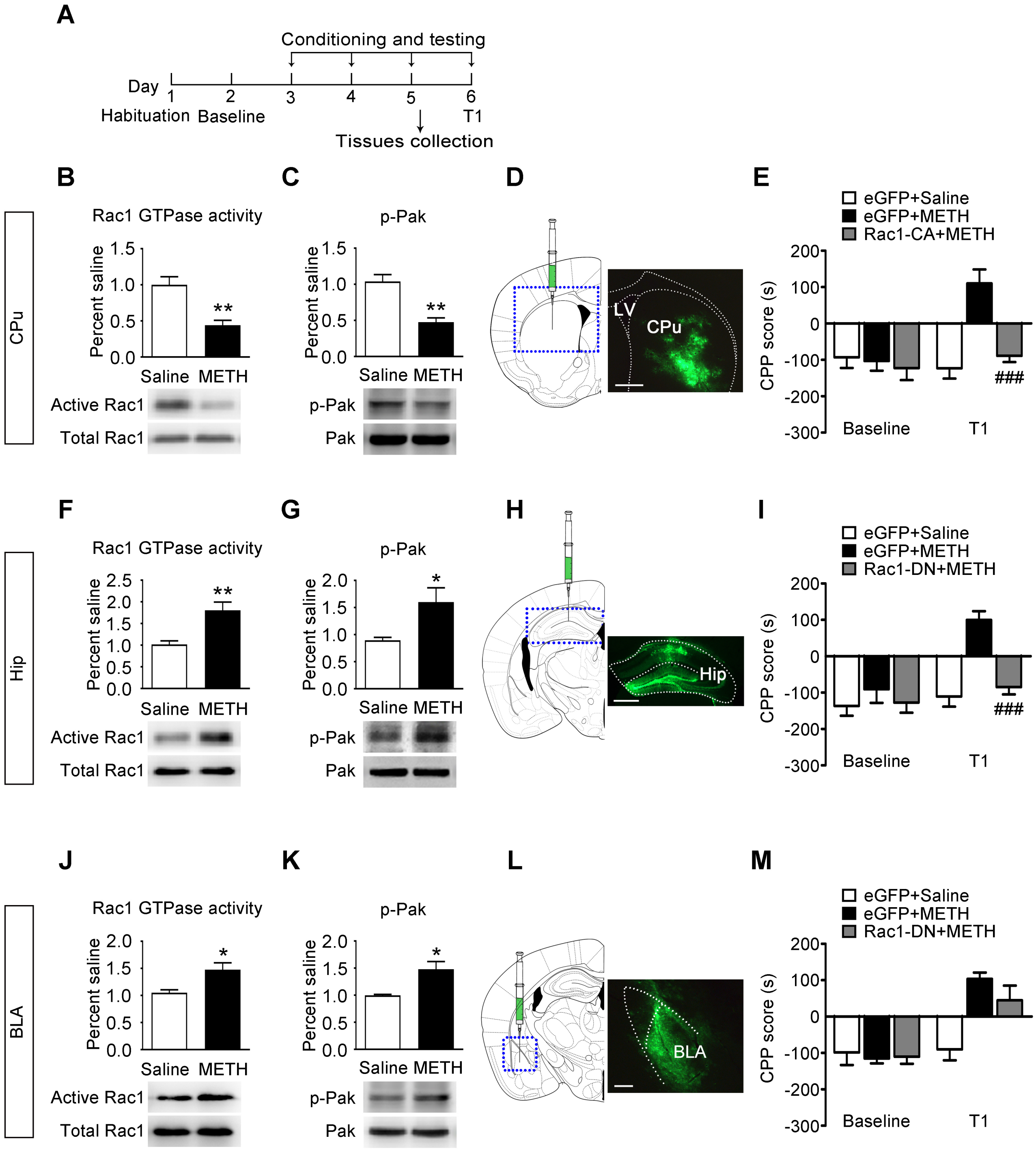

Figure S1. Region-specific role of Rac1 signalling in the METH-associated contextual memory

(A) Experimental paradigm for METH-induced conditioned place preference (CPP) and tissues collection. Mice were conditioned with METH or saline for 3 d, and each section of their brain tissues was dissected immediately after the final conditioning

training session, and the levels of Rac1 and p-Pak were investigated. **(B-C)** The activity of the Rac1 and p-Pak was decreased in the CPu. **(D)** Anatomical location of the CPu in the mice injected with a lentivirus expressing eGFP. Scale bar=500 μm. **(E)** Overexpression of Rac1-CA in the CPu attenuated METH-CPP acquisition. **(F-G)** The activity of the Rac1 and p-Pak was increased in the hippocampus. **(H)** Anatomical location of the hippocampus in the mice injected with a lentivirus expressing eGFP. Scale bar=500 μm. **(I)** Overexpression of Rac1-DN in the hippocampus attenuated METH-CPP acquisition. **(J-K)** The activity of Rac1 and p-Pak was increased in the BLA. **(L)** Anatomical location of the BLA in the mice injected with a lentivirus expressing eGFP. Scale bar=200 μm. **(M)** Overexpression of Rac1-DN in the BLA had no effect on METH-CPP acquisition. Data were analysed using a Student's t-test or one-way ANOVA followed by Bonferroni's post hoc test and presented as mean±SEM. ***p<0.001, **p<0.01, and *p<0.05 compared to the saline group; ###p<0.001, ##p<0.01, and #p<0.05 compared to the eGFP METH group. BLA: basolateral amygdala; CPu: caudate putamen; Hip: hippocampus; T1: test 1; T2: test 2.

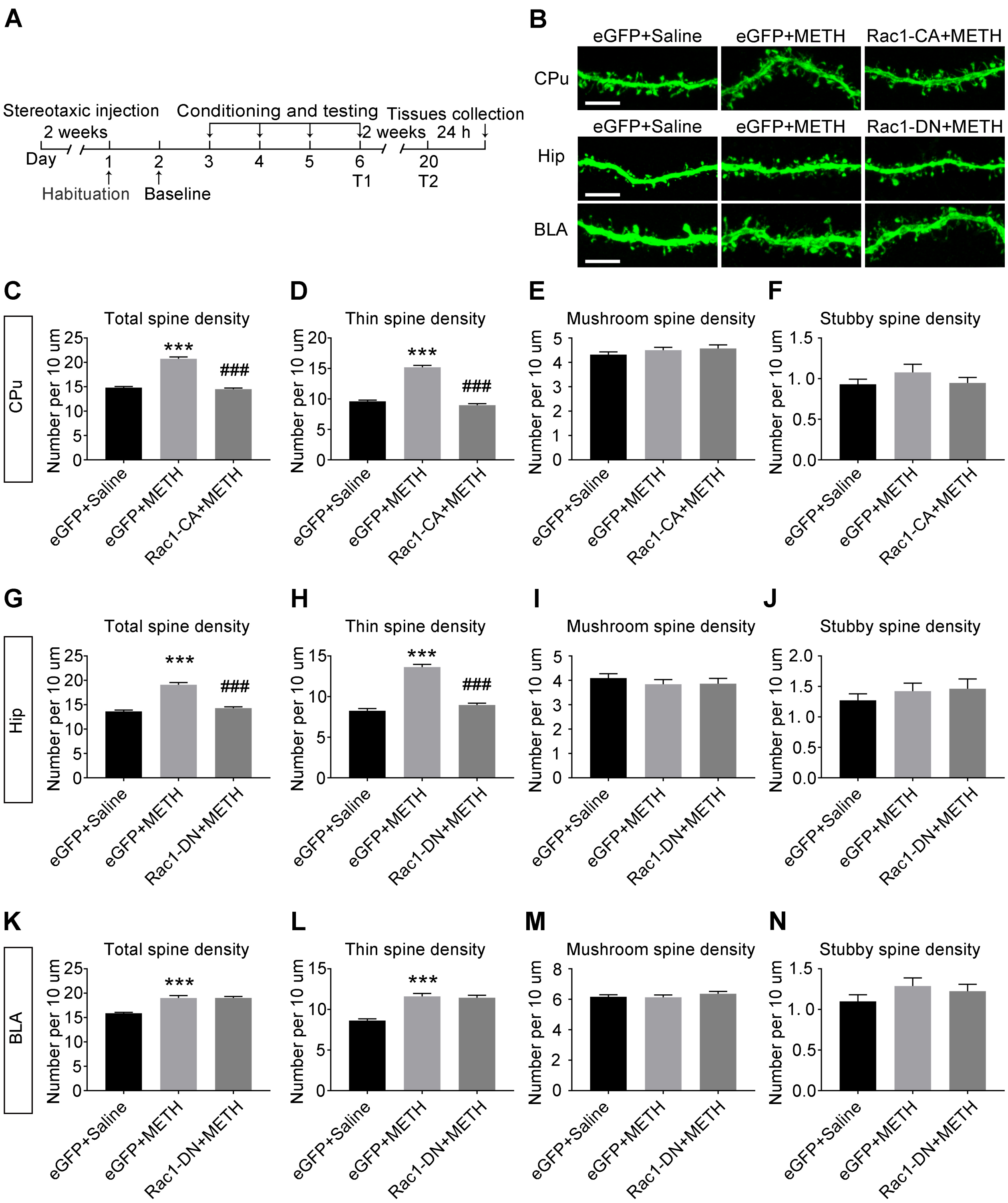

Figure S2. Effect of Rac1 signalling on METH-induced spine remodelling in the CPu, Hip and BLA

(A) Experimental design for virus injection and tissues collection. **(B)** Representative images of dendrites in all groups. Scale bar=5 μm. **(C-F)** METH significantly increased the total and thin spine density in the CPu without affecting mushroom and

stubby spine density. METH-induced increase in the spine density was reversed by Rac1-CA. **(G-J)** The increase in the total and thin spine density induced by METH in the hippocampus was reversed by Rac1-DN. **(K-N)** The total and thin spine density were increased in the BLA after METH treatment, and Rac1-DN had no effect on the spine density in the BLA. Data were analysed using one-way ANOVA followed by Bonferroni's post hoc test and presented as mean±SEM. ***$p<0.001$, **$p<0.01$, and *$p<0.05$ compared to the eGFP saline group. ###$p<0.001$, ##$p<0.01$, and #$p<0.05$ compared to the eGFP METH group. BLA: basolateral amygdala; CPu: caudate putamen; Hip: hippocampus; T1: test 1; T2: test 2.

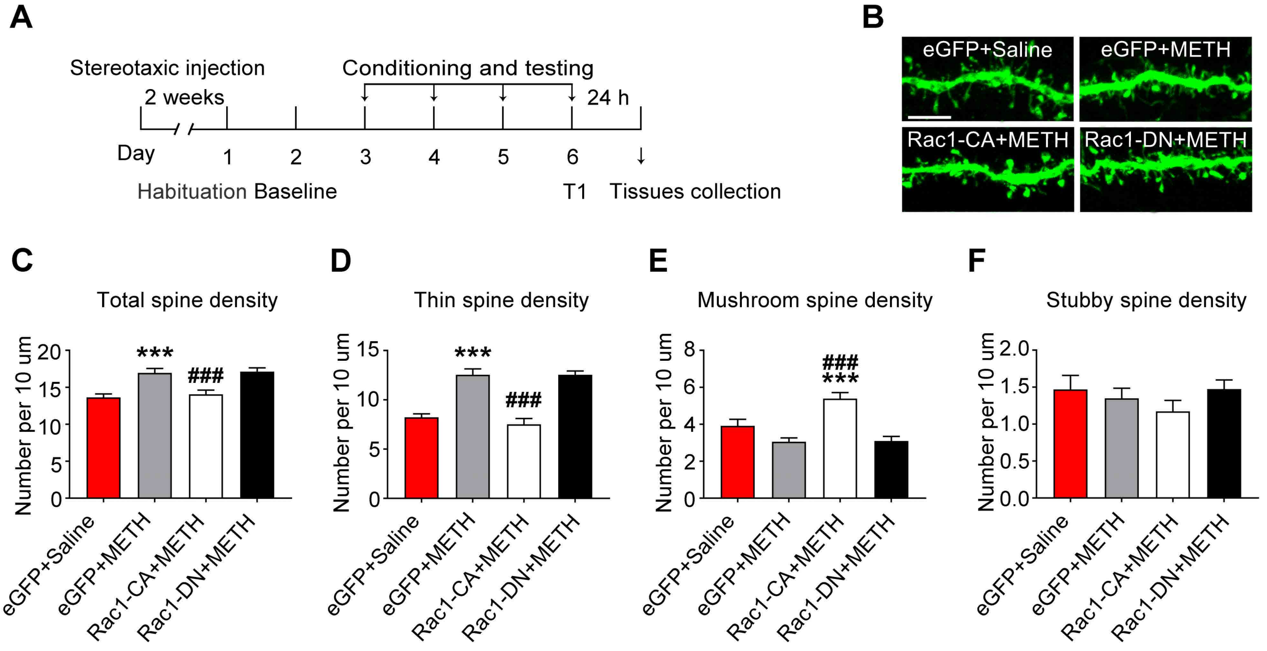

Figure S3. Decreased Rac1 signalling mediates spine remodelling during the acquisition of METH-associated contextual memory

(A) Experimental design used for the virus injection and tissues collection. **(B)** Representative images of dendritic segments in all group. Scale bar=5 μm. **(C-F)** METH increased the total and thin spine density 24 h after CPP test 1, and the METH-induced increases in the total and thin spine density were blocked by Rac1-CA. METH had no effect on mushroom and stubby spine density. Data were analysed using one-way ANOVA followed by Bonferroni's post hoc test and presented as mean±SEM. ***p<0.001, **p<0.01, and *p<0.05 compared to the eGFP saline group; ###p<0.001, ##p<0.01, and #p<0.05 compared to the eGFP METH group; T1: test 1.

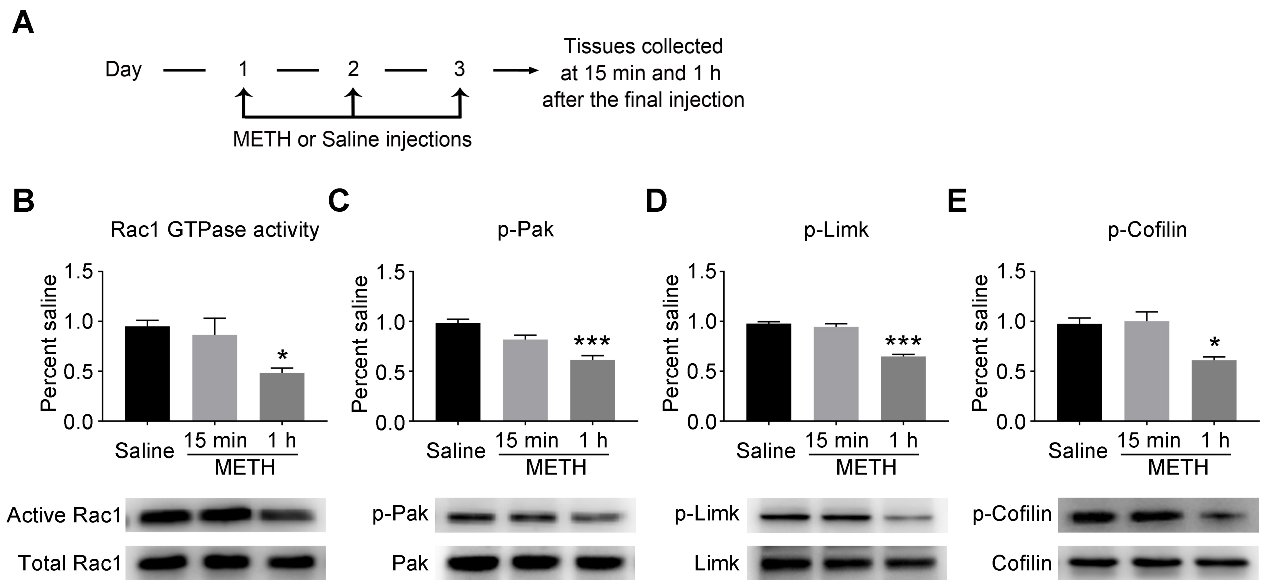

Figure S4. The Rac1 signalling was decreased after repeated METH treatment. (A) Timeline of the injections. Mice received three once-daily injections of either METH (2 mg/kg) or saline in their home cage, and then, the NAc tissues were collected 15 min or 1 h following the final injection. **(B-E)** The activity of Rac1, p-Pak, p-Limk, and p-Cofilin in the NAc was decreased 1 h after the final injection, without significant alterations 15 min after the final injection. Data were analysed using one-way ANOVA followed by Bonferroni's post hoc test and presented as mean±SEM. ***$p<0.001$, **$p<0.01$, and *$p<0.05$ compared to the saline group.

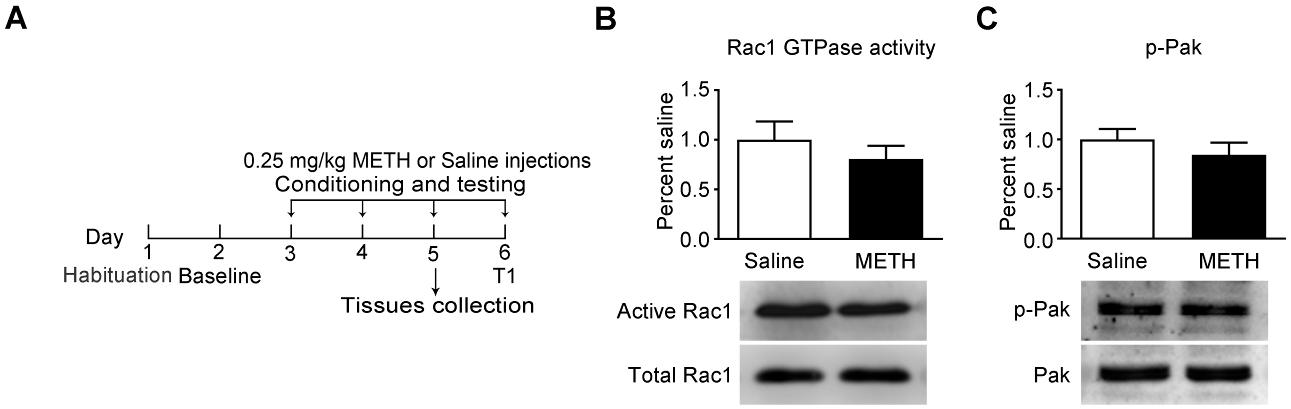

Figure S5. The Rac1 signalling was unchanged after repeated low-dose treatment with METH.

(A) Experimental paradigm for METH-induced conditioned place preference (CPP). Mice were conditioned with METH (0.25 mg/kg) or saline, and then, the NAc tissues were collected immediately after the final METH conditioning training session. **(B-C)** Compared to the saline-treated mice, the activity of Rac1 and p-Pak in METH-treated (0.25 mg/kg) mice was unchanged. Data were analysed using a Student's t-test and presented as mean±SEM. T1: test 1.
